# Litter chemistry explains contrasting feeding preferences of bacteria, fungi, and higher plants

**DOI:** 10.1038/s41598-017-09145-w

**Published:** 2017-08-23

**Authors:** Giuliano Bonanomi, Gaspare Cesarano, Nadia Lombardi, Riccardo Motti, Felice Scala, Stefano Mazzoleni, Guido Incerti

**Affiliations:** 1 Dipartimento di Agraria, University of Naples Federico II, via Università 100, 80055 Portici (NA), Italy; 2 Department of Agri-Food, Animal and Environmental Sciences, University of Udine, via delle Scienze 206, 33100 Udine, Italy

## Abstract

Litter decomposition provides a continuous flow of organic carbon and nutrients that affects plant development and the structure of decomposer communities. Aim of this study was to distinguish the feeding preferences of microbes and plants in relation to litter chemistry. We characterized 36 litter types by ^13^C-CPMAS NMR spectroscopy and tested these materials on 6 bacteria, 6 fungi, and 14 target plants. Undecomposed litter acted as a carbon source for most of the saprophytic microbes, although with a large variability across litter types, severely inhibiting root growth. An opposite response was found for aged litter that largely inhibited microbial growth, but had neutral or stimulatory effects on root proliferation. ^13^C-CPMAS NMR revealed that restricted resonance intervals within the alkyl C, methoxyl C, *O*-alkyl C and di-*O*-alkyl C spectral regions are crucial for understanding litter effects. Root growth, in contrast to microbes, was negatively affected by labile C sources but positively associated with signals related to plant tissue lignification. Our study showed that plant litter has specific and contrasting effects on bacteria, fungi and higher plants, highlighting that, in order to understand the effects of plant detritus on ecosystem structure and functionality, different microbial food web components should be simultaneously investigated.

## Introduction

Plant litterfall delivered to soil system represents a large fraction of terrestrial ecosystem primary productivity^1, 2^. Freshly fallen litter is subject to decomposition by a large diversity of microbial saprotrophs and invertebrate detritivores that co-occur with plant residues decomposition forming complex food webs^3^. Moreover, litter decay is a key process for terrestrial ecosystem functioning, serving as a main source of nutrients and organic compounds that sustain plant productivity, contribute to soil organic matter formation, and affect most biogeochemical cycles^4^. Decades of intensive ecological studies have well established the role of climatic conditions (mainly temperature, moisture and UV radiation^5, 6^), on litter decomposition dynamics, which also depend intrinsically on the molecular composition of plant residues^7, 8^. Concerning biotic factors, many studies have described the community structure and composition of invertebrate detritivores^9^ and bacterial^10^ and fungal^11^ decomposers, while less attention has been given to their functional role and effect on litter decomposition dynamics^3^.

Plant litter is a primary determinant of the community structure of plants^12, 13^ and microbes^10^. Several studies have demonstrated that litter can either limit or promote plant development by affecting species establishment and growth through a variety of mechanisms, such as shading^14^, mechanical impediment^15^ and release of allelochemicals during the decomposition process^16, 17^. Three recent surveys, based on 21^18^, 64^19^ and 65^20^ different plant materials, showed that litter effects can largely vary in magnitude and direction in relation to the molecular composition of plant residues, which in turn depend on plant functional type and litter decomposition stage. Indeed, decomposing plant materials can release both essential mineral nutrients^21^ and labile inhibitory compounds^17^. On this basis, two mutually non-exclusive hypotheses have been proposed to explain plant litter inhibitory effects: nitrogen (N) immobilization by microbial competition^22, 23^ and phytotoxicity by labile organic compounds^19, 24^. According to the first hypothesis, at low N availability, as in decaying plant residues with a high C:N ratio, saprophytic microbes would compete with plants for this limiting resource^23, 25^. In contrast, the second hypothesis sustains a direct negative effect of a wide array of inhibitory compounds, early released by decomposing litter, including short-chain organic acids^26^, tannins^27^ and phenols^28^. In this regard, litter decay dynamics are of utmost importance for the allelopathic impact of plant litter. Indeed, the relative abundance and activity of phytotoxic compounds dynamically change during litter decay as a result of chemical-physical transformations and biological processes driven by microorganisms^24, 28^. While a rapid degradation of most allelochemicals into non-toxic molecules has often been reported^19, 29, 30^, the persistence of extracellular fragmented plant DNA in decomposed litter can produce species-specific effects at medium-long term, ranging from inhibition of conspecifics to stimulation of heterospecifics^31^.

In addition to complex effects on plant development, litter dynamics critically affect the structure and composition of decomposer communities. A large number of studies which describe the successional dynamics of bacterial^10^ and especially fungal communities^32–34^ that occur during litter decomposition, report several compositional shifts and a general increase of microbial diversity at decreasing litter quality. Several mechanisms have been proposed to explain the observed patterns, including limitation of spore dispersal, inter-specific competition^11^, time constraints for fruiting body development^35^, grazing pressure by arthropods^36^, and shifts of substrate suitability owing to litter biochemical dynamics^37, 38^. In this context, it is relevant that heterotrophic saprotrophic microbes, by using plant litter as a source of energy and nutrients, progressively modify the biochemical quality of organic substrates^39, 40^ which, in turn, affects microbial turnover, community composition and diversity. For example, the rapid disappearance of weak parasites and “sugar fungi” replaced by cellulolytic and ligninolytic fungi, (a well recognized successional pattern^11, 32^), has been associated with the rapid depletion of labile C compounds and the concomitant relative increase of cellulose and lignin content in litter^41^. In addition, according to Prescott^42^, stable organic matter would be produced by microbial and biochemical transformation of litter materials into novel recalcitrant compounds, rather than by selective preservation of the pre-existing resistant fraction. Several studies based on throughput analytical techniques such as pyrolysis-gas chromatography/mass spectrometry^43^, near infrared reflectance spectroscopy^44^, and ^13^C-CPMAS NMR spectroscopy^2^ have clarified major molecular traits^45^ and changes^46, 47^ corresponding to litter ageing. These include a rapid reduction of aminoacids, polypeptides, *O*-alkyl and di-*O*-alkyl fractions corresponding to carbohydrates, and a relative increase of lignin content and the associated methoxyl and N-alkyl compounds. Correspondingly, litter suitability for the growth of opportunistic saprophytic fungi progressively decreases with litter age^40^.

Taken together, such evidence indicates litter molecular composition is a main driver of bacteria, fungi and higher plant development on litter materials. Previous studies have separately tested litter effects on higher plants^18–20, 25^, bacteria^10^, or fungi^40^, while tests simultaneously targeting representatives of all these microbial food web components over the same materials have not yet been reported. In addition, most previous evidence relies on over-simplified systems including a single or at most few sensitive target species of little ecological relevance. Such study conditions clearly do not match those in nature, where plant litter simultaneously affects different, highly diverse trophic levels. The strength of the conclusions that can be drawn at this stage has thus been limited. Moreover, the use of single-species bioassay prevents the assessment of species-specific responses possibly related to differences in sensitivity and in functional traits, even within the same trophic level. Many field and greenhouse experiments limited to plants, however, report that a thick litter layer mostly hampers the establishment of short-lived, small seeded species^48, 49^, owing to the scarce penetration capability of small seedlings or to the requirements of light for germination of small seeds^50^. Surprisingly, in this context, the hypothesis of a higher sensitivity to litter inhibitory effects in small-seeded seedling compared to large-seeded one has not yet been tested.

In this study, we combined litter characterization by ^13^C-cross-polarization magic angle spinning nuclear magnetic resonance (^13^C-CPMAS NMR) spectroscopy^2^, proximate analysis^51^ and C and N content determination, with a multi-species bioassay. In particular, the effect of 36 litter types spanning a wide range of biochemical quality was assessed on 14 target plants, 6 fungi and 6 bacteria species. We expect a progressive decrease of bacterial and fungal growth with litter age. Differently, for plants, we expect a shift of litter effects on root growth during litter decay, from inhibitory to enhancing, in relation to changes in litter chemistry. We also explicitly hypothesize a negative association between seed size and sensitivity to litter inhibitory effects, expecting that seedlings of small-seeded plants are more prone to litter inhibitory effects compared to seedlings of large-seeded plants. Finally, based on the finer molecular resolution of ^13^C-CPMAS NMR spectroscopy compared to traditional indices based on C-to-N, we expect that specific spectral data are associated to litter functional quality in relation to bioassay results.

## Results

### Effects of litter materials

**Figure 1:**
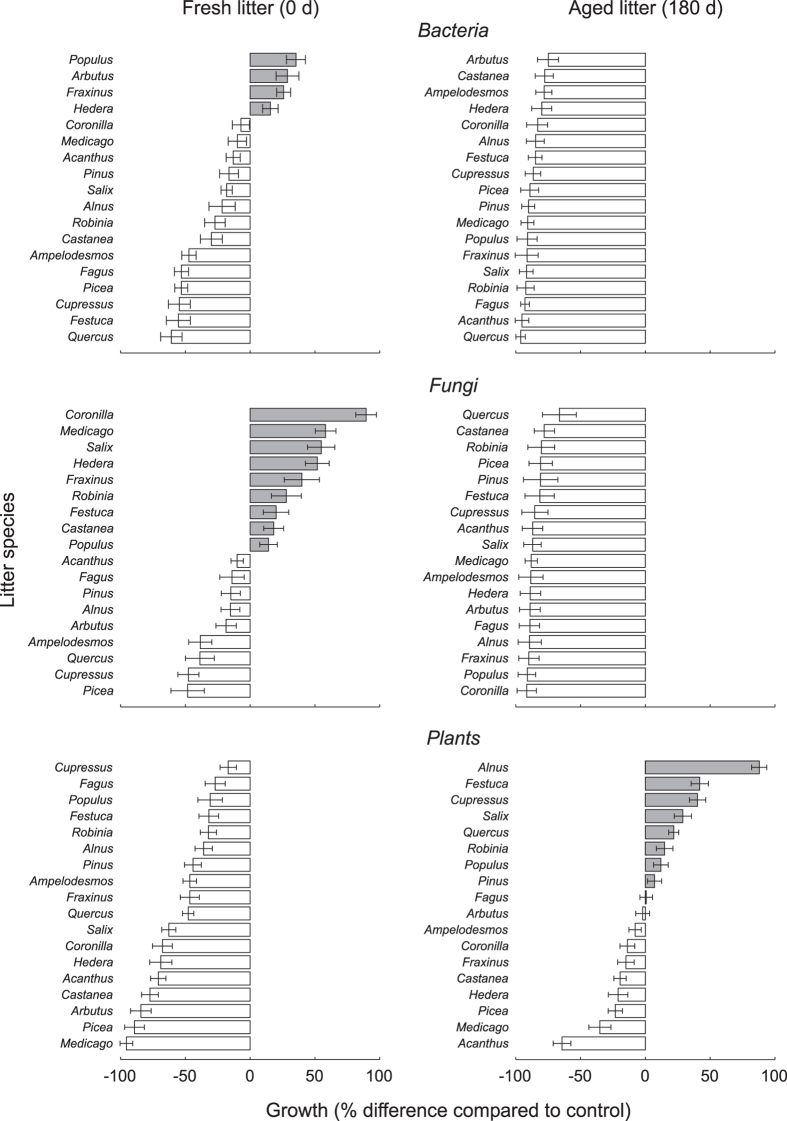
Effects of 18 different litter materials, either undecomposed (left) or after 180 days of decomposition (right), on the growth of selected bacteria (top) and fungi (center) and higher plants (bottom). Growth is expressed as percent difference compared to the controls (Nutrient Broth, and PDA and water for bacteria, fungi and plants, respectively). White and gray bars indicate inhibitory and stimulatory effects, respectively. For each bar, data refer to mean ± standard error of 14, 6 and 6 target species for plants, bacteria and fungi, respectively.

**Figure 2:**
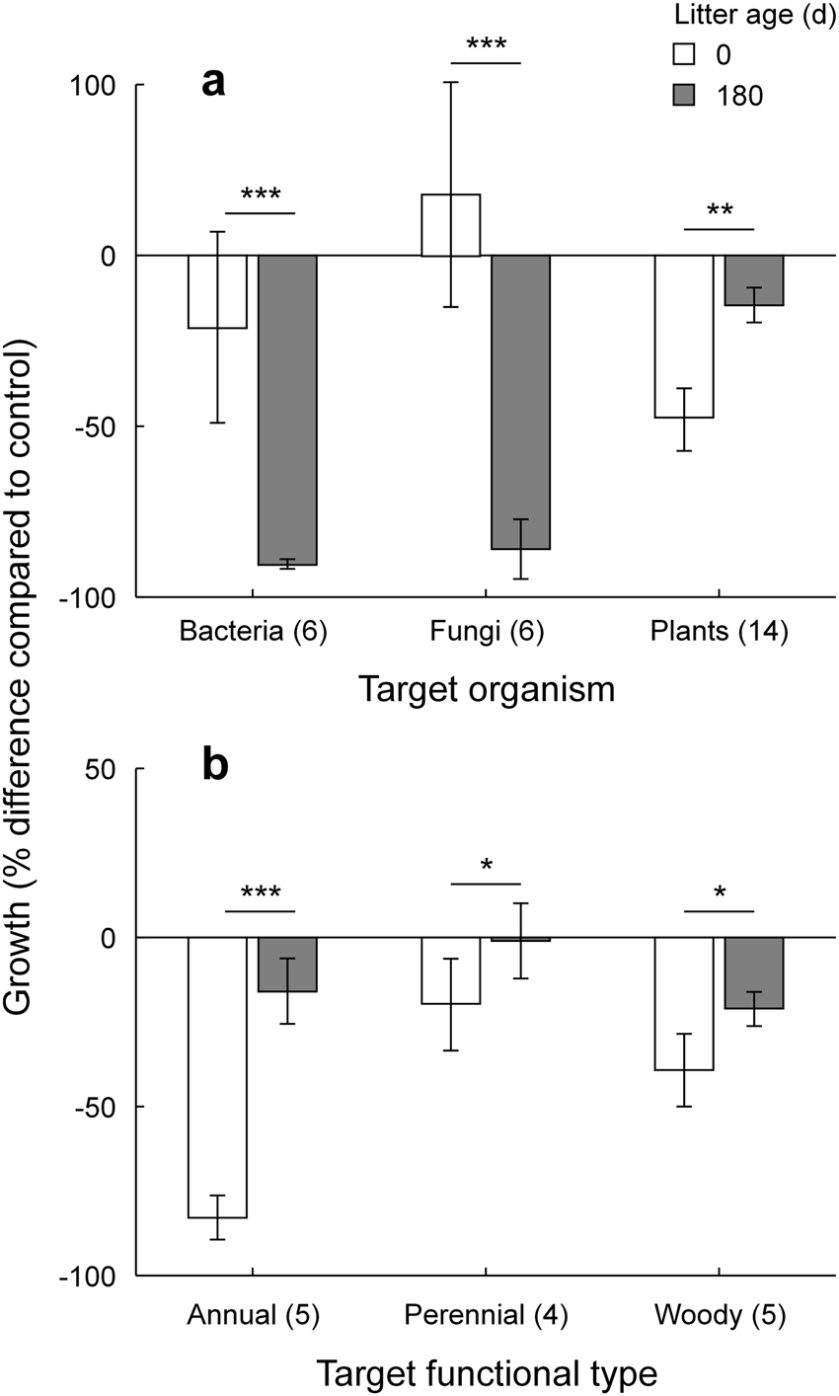
Growth response of different target organisms (**a**) and plant functional types (**b**) to either undecomposed (0 d) or decomposing (180 d) litter. Growth is expressed as percent difference compared to the controls (Nutrient Broth, PDA, and water for bacteria, fungi, and plants, respectively). For each bar, data refer to mean ± standard deviation of different target species (N in brackets) over18 litter types. Asterisks indicate within-group statistically significant differences (***p < 0.001; **p < 0.01; *p < 0.05), according to Tuckey’s HSD post-hoc tests for the interactive effects of litter age and target organisms (**a**) and litter age and plant functional type (**b**) from corresponding GLMMs models in Table 2.

The effect of litter on plants, bacteria and fungi was largely determined by litter type and age (Fig. 1). Bacteria response over undecomposed litter showed on average an inhibitory effect compared to the control (Fig. 1). However, the effect largely varied according to litter type, being positive for *Populus*, *Arbutus*, *Fraxinus*, and *Hedera* litters, markedly inhibitory for *Quercus*, *Festuca*, *Cupressus*, *Picea* and *Fagus* materials, and showing an intermediate pattern for the remaining species (Fig. 1). Fungi, on average, showed higher growth on undecomposed litter compared to the control (+18%). Consistent with bacteria response, also for fungi the effect of undecomposed material was largely affected by litter type. A positive response was found for 9 litter types (e.g. *Coronilla*, *Medicago*, *Salix*) and a moderate inhibition over the remaining 9 materials (Fig. 1). Interestingly, both bacteria and fungi showed a response pattern to litter age opposite to that of higher plants (Figs 1 and 2). In particular, bacteria and fungi showed a significantly higher growth on undecomposed compared with 180-days old litter (Fig. 2a), corresponding to a remarkable growth inhibition (>50%) over all aged litter types (Fig. 1).

Over undecomposed litter, plants showed on average a root inhibition of 48% compared with control. While all undecomposed materials showed a significant inhibitory effect on target plant species (Fig. 1), the response varied according to litter type, being very high for *Medicago*, *Picea*, and *Arbutus* material, relatively low for *Populus*, *Fagus* and *Cupressus* litter and intermediate for the remaining plant residues (Fig. 1). Decomposition for 180 days greatly reduced the inhibitory effect, with 9 litter types that stimulated root growth of the target species compared to untreated control (Fig. 1). The only exceptions were *Acanthus mollis* and *Medicago sativa* materials that were still highly inhibitory after 180 days of decomposition (Fig. 1). Plant functional groups showed a different response to litter, with annuals species being more inhibited on undecomposed litter compared with perennial and woody species (Fig. 2b).

### Species-specific sensitivity to litter effects in plants, bacteria and fungi

Overall, all bacterial and fungal species grew well over fresh, undecomposed litter compared with aged substrates (Fig. S1a,b; Table 1). Among bacteria, *Rhizobium* was the most inhibited over aged litter while *Bacillus* showed the highest growth over undecomposed substrates (Fig. S1a). Concerning fungi, all species thrived over fresh litter, with very high growth for *Mucor* and *Trichoderma* (Fig. S1b). Differently, over aged substrates, only *Trichoderma* showed a relatively positive performance, while the remaining five species were severely inhibited, as compared to the control over PDA as well as the performance over undecomposed litter (Fig. S1b).

**Table 1:** Summary of the Generalized Linear Mixed Models (GLMM) testing for main and 2^nd^ order interactive effects of litter species (random effect) and age (fixed effect, either 0 or 180 days of decomposition) on bioassay results (i.e. growth of plants, fungi and bacteria, expressed as percentage of the untreated control).

	Effect type	SS	df	MS	F	*p*
***All data pooled***						
Litter species (L)	Random	766411	17	45083	3.24	0.0058
Litter age (A)	Fixed	212583	1	212583	26.09	0.0001
Target organism group (O)	Fixed	4789183	2	2394591	190.77	<0.0001
L × A	Random	207740	17	12220	2.00	0.0412
L × O	Random	426784	34	12552	1.95	0.0273
A × O	Fixed	2488776	2	1244388	193.81	<0.0001
***Bacteria***						
Litter species (L)	Random	219385	17	12905	2.33	0.0459
Litter age (A)	Fixed	236545	1	236545	15.60	0.0049
Target species (T)	Random	329809	5	65962	5.38	0.0425
L × A	Random	91187	17	5364	2.33	0.0057
L × T	Random	209600	85	2466	1.07	0.3723
A × T	Random	60483	5	12097	5.27	0.0003
***Fungi***						
Litter species (L)	Random	358819	17	21107	2.40	0.0353
Litter age (A)	Fixed	962182	1	962182	44.51	0.0001
Target species (T)	Random	412704	5	82541	5.33	0.0406
L × A	Random	141914	17	8348	4.77	<0.0001
L × T	Random	188262	85	2215	1.27	0.1397
A × T	Random	75104	5	15021	8.58	<0.0001
***Plants***						
Litter species (L)	Random	821469	17	48322	9.22	<0.0001
Litter age (A)	Fixed	1308116	1	1308116	16.95	0.0012
Target species (T)	Random	2604705	13	200362	2.57	0.0492
L × A	Random	150299	17	8841	2.00	0.0123
L × T	Random	1153846	221	5221	1.19	0.1019
A × T	Random	1003151	13	77165	17.54	<0.0001
***Plant functional types***						
Litter species (L)	Random	853545	17	50209	6.74	0.0003
Litter age (A)	Fixed	1204105	1	1204105	252.35	<0.0001
Target functional type (F)	Fixed	1008085	2	504042	65.84	<0.0001
L × A	Random	162235	17	9543	1.93	0.0490
L × F	Random	260288	34	7656	1.54	0.1060
A × F	Fixed	721938	2	360969	72.69	<0.0001

GLMMs were tested for all data pooled, including a fixed effect of the group of organisms (3 levels), and separately for plants, fungi and bacteria, including a random effect of target species. In the case of plants, a further GLMM was tested, including the effect of target functional type (3 levels: annual, perennial and woody).

**Figure 3:**
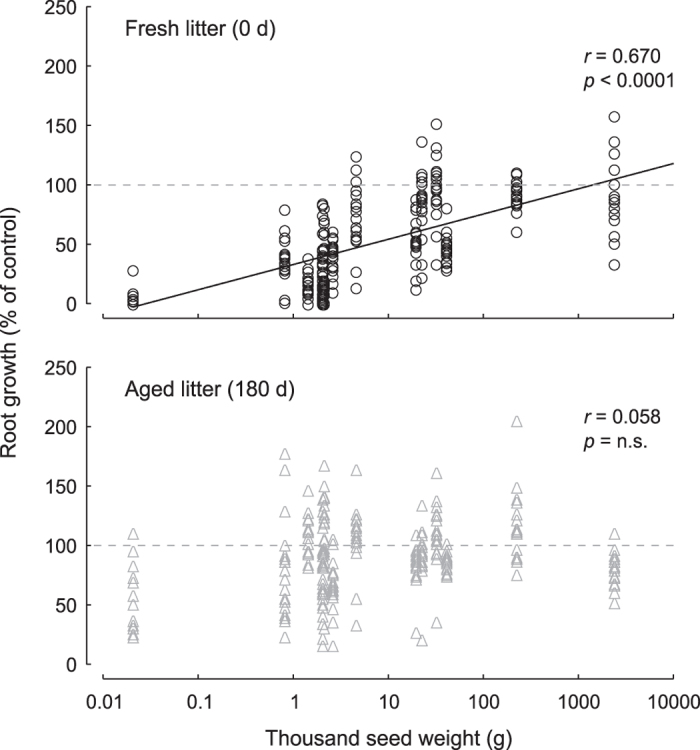
Relationships between seed weight and root growth of 14 target plants sown over 36 litter materials, either undecomposed (top) or decomposed for 180 days (bottom). Note logarithmic scale for seed weight. Data refer to mean growth of 10 replicates for each combination of target plant, litter species and age, as compared to the control (gray horizontal dashed line). Logarithmic fit is also reported in each panel, and symbolized with either continuous or dotted black line according to significant or not significant result, respectively.

Concerning higher plants, species-specific responses to litter treatments were observed (Fig. S1c). Over undecomposed litter, the five annual species were the most inhibited together with the deciduous tree *Populus* (Fig. S1c), with *Arabidopsis*, *Lycopersicon*, *Trifolium* and *Lepidium* almost completely inhibited over fresh, undecomposed litter. In contrast, some perennial and woody species were slightly affected compared to the untreated control (Fig. S1c), also showing, in the cases of *Hedera*, *Pinus* and *Quercus*, a not significantly different response to fresh and aged litter. Concerning root growth over aged litter, *Arabidopsis* were the most inhibited species (Fig. S1c) but the difference among target plants was largely reduced, with most species showing minor variations, of both signs, compared to the untreated control (Fig. S1c). Interestingly, seedling root growth over undecomposed litter was positively associated with seed weight, while such relationships did not hold over aged litter (Fig. 3).

### Litter chemistry and target species sensitivity

Litter proximate chemical parameters showed a contrasting pattern of correlations with the response of microbes and plants (Table 2). In particular, labile C was positively associated with fungal growth, and negatively with root growth of annual, perennial, and woody plant species (Table 2). Cellulose and N content did not show significant relationships with the response of target organisms (Table 2). Finally, C/N ratio was negatively and positively correlated with growth of annual plant and fungi, respectively (Table﻿ 2).

**Table 2:** Correlation (Pearson’s r) between bioassay results averaged for different target organisms (i.e. growth of bacteria, fungi and plant functional types) and biochemical quality of the 36 litter types assessed by proximate chemical parameters and ^13^C-CPMAS NMR data.

Litter biochemical quality	Microbes		Higher plants			
	Bacteria	Fungi	Annual	Perennial	Woody	All plants
*Chemical parameters*						
Labile C (%)	0.36	**0**.**52**	***−0***.***59***	**−0**.**53**	**−0**.**45**	**−0**.**50**
Cellulose (%)	0.11	0.17	−0.11	0.15	0.12	−0.01
N content (%)	−0.09	−0.14	0.16	−0.05	−0.21	−0.02
C/N ratio	0.27	**0**.**44**	**−0**.**49**	−0.14	−0.08	−0.26
^13^ *C-CPMAS NMR parameters*						
Carboxylic C: 161–190 p.p.m.	−0.06	−0.10	0.21	−0.20	−0.22	−0.05
O-sub. aromatic C: 141–160 p.p.m.	−0.16	−0.13	0.01	0.35	**0**.**45**	0.28
H- & C-sub. aromatic C: 111–140 p.p.m.	−0.22	−0.18	0.17	0.30	0.33	0.26
di-O-alkyl C: 91–110 p.p.m.	0.13	0.35	−0.30	0.29	0.21	0.03
O-alkyl C: 61–90 p.p.m.	0.22	**0**.**48**	−0.38	0.17	0.08	−0.08
Methoxyl and N-alkyl C: 46–60 p.p.m.	−0.30	**−0**.**51**	**0**.**52**	0.08	0.07	0.25
Alkyl C: 0–45 p.p.m.	−0.10	−0.36	0.23	−0.31	−0.21	−0.06
Alkyl C/O-alkyl C ratio	−0.15	−0.40	0.28	−0.28	−0.18	−0.02
CC/MC ratio*	0.00	−0.15	0.07	−0.08	−0.05	0.00

Bold indicates statistically significant values of *r* (p < 0.025, after controlling for multiple comparison according to Benjamini and Hochberg^74^).

*: CC ⁄ MC: O-alkyl C/methoxyl and N-alkyl C ratio.

Considering ^13^C-CPMAS NMR reference regions and growth data pooled for either bacteria or fungal species, no significant correlations were found between litter molecular composition and microbial growth (Table 2). The only two exceptions were the *O*-alkyl C and methoxyl C litter fractions, positively and negatively related to fungal growth, respectively (Table 2). Considering higher plant responses, we observed a general pattern of non-significant relationships between seedling root growth and C-types corresponding to spectral regions, with the exceptions of annual and woody species, positively related to methoxyl C and *O*-substituted aromatic C, respectively (Table 2). The widely used alkyl C/*O*-alkyl C and CC/MC ratios were not significantly related to bacteria, fungal and plant growth (Table 2).

**Figure 4:**
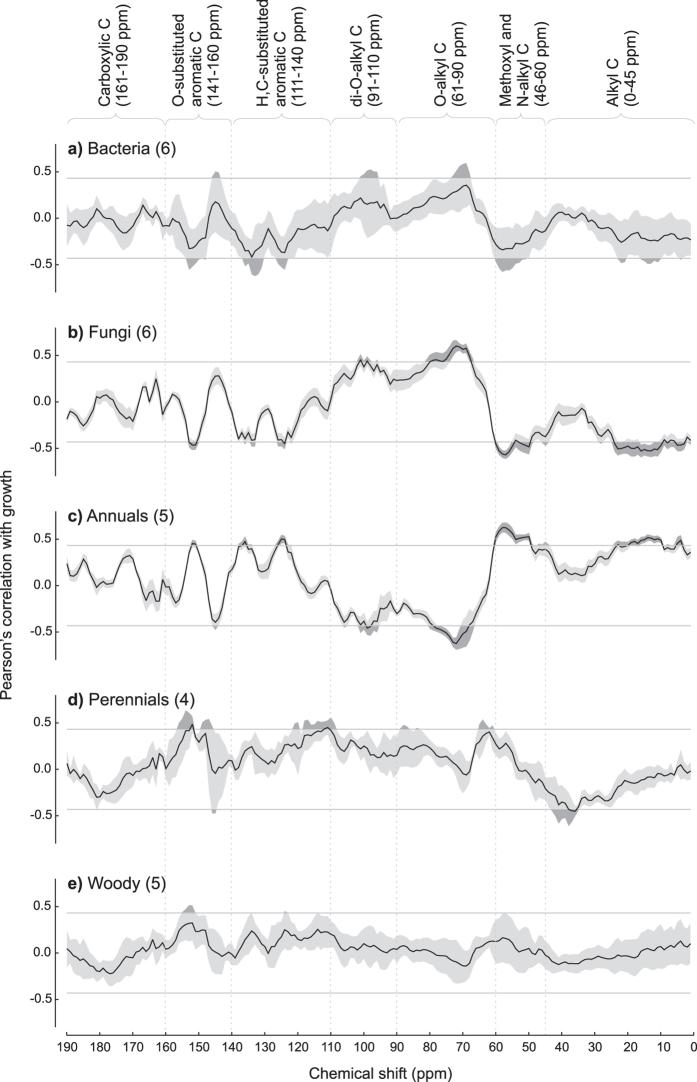
Correlation profiles between the growth of different organisms (bacteria, fungi, annual plants, perennial plants, woody plants) over 36 different litter materials (i.e. fresh and decomposed leaves from 18 species) and ^13^C-CPMAS NMR spectral signals of the same materials. In each panel, vertical dashed lines refer to main classes of organic C. Horizontal gray lines indicate threshold values for significant correlation (*p* < 0.01 after controlling for multiple comparison according to Benjamini and Hochberg^74^). Data refer to Pearsons’ *r*, with mean (solid line) and 95% confidence interval (filled bands) calculated over a different number of target species within each organism type (in brackets). Significant *r* values are highlighted in dark gray.

An extensive correlation analysis of all signals from the ^13^C-CPMAS NMR litter spectra with the response of the 26 target species on the same materials showed an interesting pattern of association between the growth of target organisms, and some restricted spectral regions (Fig. 4). In the case of bacteria, the relationships between litter chemistry and species response along the spectrum, considering data averaged over the six target species, did not show a significant correlation (Fig. 4a). However, such a profile showed high inter-specific variation (Fig. 4a), with restricted spectral regions significantly related to the growth of different species (Supplementary Table S1). Among these, we observed negative correlations with spectral signals resonating in the regions 4–14 ppm (for *Bacilus* and *Escherichia*), 53–60 ppm (*Bacillus*, *Escherichia*, *Lysobacter* and *Pseudomonas*), 123–126 ppm (*Escherichia*, *Erwinia* and *Pseudomonas*), 132–138 ppm (i.e. *Bacillus*, *Escherichia*, *Erwinia*, and *Pseudomonas*), 151–153 ppm (*Bacillus*, *Escherichia*, *Erwinia*, *Pseudomonas*), and positive association with signals in the 68–78 ppm region (i.e. *Bacillus*, *Escherichia*, *Erwinia* and *Pseudomonas*) (Supplementary Table S1). Further, single species were positively associated with specific signals, as in the cases of *Bacillus* and *Lysobacter* with the signals at 97–101 ppm and 144–146 ppm, respectively. Finally, *Rhizobium* did not show significant correlations along the whole spectrum (Supplementary Table S1).

The six target fungi showed an exceptionally similar pattern of correlation with litter chemistry defined by ^13^C CPMAS NMR (Fig. 4b). All species showed positive correlations with the signals resonating within the 67–82 ppm and 96–106 ppm regions, and negative with those at 3–24 ppm, 45–60 ppm, 122–126 ppm, 132–138 ppm and 151–153 ppm (Fig. 4b), with the exceptions of single species showing marginally significant correlation with specific signals (Supplementary Table S2), as in the case of *Ganoderma* (122–126 and 151–153 ppm), *Trichoderma* (3–24 and 132–138 ppm) and *Umbelopsis* and *Mucor* (95–105 ppm).

In the case of higher plant growth, a different pattern of association with litter chemistry was observed for annual, perennial and woody species (Fig. 4c–e). Annual plants showed a very similar response among the five species (Supplementary Table S3) with an outstanding opposite behaviour compared to fungi (Fig. 4b,c). In particular, all annual plants were positively associated with the signals resonating within the 3–24 ppm, 45–60 ppm, 122–126 ppm, 133–138 ppm and 151–153 ppm, and negatively with those at 67–80 ppm and 96–106 ppm regions (Fig. 4c, Supplementary Table S3). The only exceptions were *Lepidium* and *Trifolium*, showing marginally significant correlations with the signals at 133–138 and 151–153 ppm (Supplementary Table S3). Root growth of perennial species was positively correlated with the signals resonating within the 61–65 ppm, 110–120 ppm and 145–155 ppm regions, and negatively with those within the 35–42 ppm region (Fig. 4d). However, response differences among perennial species were recorded, as in the case of *Acanthus* growth, not related with the 145–155 ppm region, and *Ampelodesmos* and *Hedera*, only marginally associated with the 61–65 ppm and 35–42 ppm regions (Supplementary Table S4). Finally, woody plant species showed a general trend of non-significant correlation between root growth and litter quality defined by ^13^C NMR (Fig. 4e), with high inter-specific differences (Supplementary Table S5). The only spectral region associated with root growth included the signals resonating at 151–155 ppm, positively correlated to root growth for *Quercus* and *Robinia*, and marginally related for *Populus*, but unrelated to *Alnus* and *Pinus* responses. *Robinia* growth was also positively associated with two single signals, resonating at 114 pm and 134 ppm, respectively (Supplementary Table S5).

**Figure 5:**
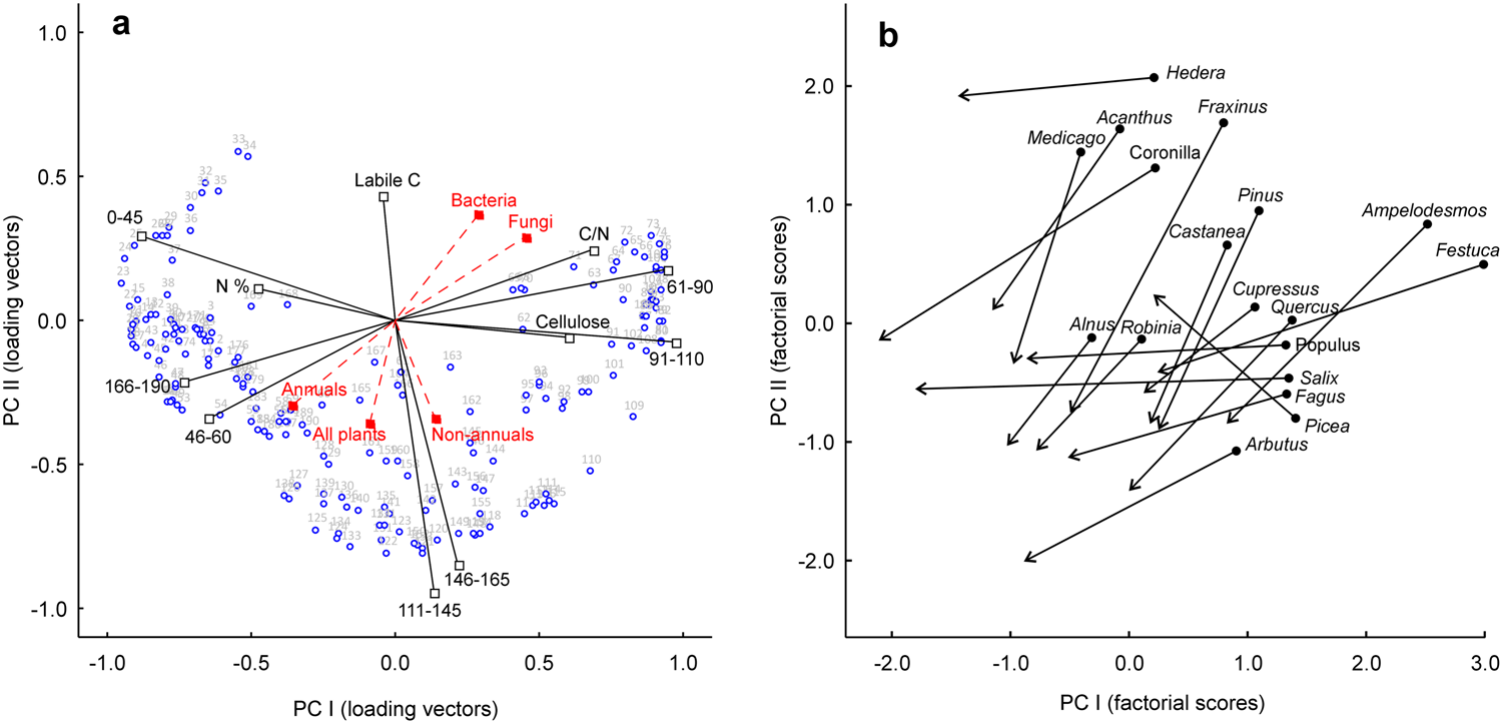
Principal component analysis of ^13^C NMR spectral signals recorded in 36 litter types (i.e. fresh and decomposed leaf materials from 18 plant species) in relation to growth response of plants and microbes and litter biochemical quality and age. (**a**) Loading vectors of spectral signals. Litter biochemical parameters and reference spectral regions (black vectors), as well as growth response of target organisms, averaged for bacteria, fungi, and annual, non-annual, and all plants (red vectors) are plotted as supplementary variables (Legendre and Legendre^75^). (**b**) Factorial scores of litter types, with decomposition trajectories between 0 and 180 days.

Principal component analysis (PCA) provided satisfactory ordination of the ^13^C-CPMAS NMR spectral signals across the 36 litter types, with the first four eigenvalues accounting for 93.2% (52.1%, 22.3%, 13.2%, and 5.6%) of the total variance, respectively. The bi-dimensional space defined by the first two components provided a synthetic picture of the litter chemistry-dependent effects of treatments on the target species responses (Fig. 5). It confirmed the above-described relationships between the relative abundance of different organic C types in the litters and the growth response of bacteria, fungi and plant functional types over such materials, based on the loading vectors of ^13^C-NMR signals measured in the litter materials (i.e. relative abundance of each signal and how they relate to the PC axes) and those of supplementary variables, including target species response and litter proximate parameters (Fig. 5a). In the same bi-dimensional space, the trajectories of the litter materials based on their factorial scores (Fig. 5b) reflected the chemical changes occurring during the decomposition process as defined by spectral data.

## Discussion

In this work, we show that plant litter has an age-dependent, contrasting effect on the growth of bacteria, fungi and higher plants. In particular, undecomposed litter has a major inhibitory effect on plants, but promotes bacterial and especially fungal growth, although the effect on microbial response largely varied according to litter type. On the contrary, as decomposition proceeds, litter becomes progressively more suitable for plant growth, while bacterial and fungal responses are largely inhibited. This is clearly related to biochemical quality shifts in decomposing materials, with a progressive depletion of easily degradable C sources and enrichment of recalcitrant compounds. Our results demonstrate that defining litter chemistry by ^13^C-CPMAS NMR provides useful insight for characterizing the substrate preference of bacteria, fungi and higher plants.

### Litter effects on higher plants

We found that undecomposed litter caused a severe inhibition on root growth of most target plants. The prevalence of inhibitory effects by undecomposed litter recorded in this survey is consistent with previous studies carried out in different ecosystems^18, 20, 25, 52, 53^. Thus, root inhibition by undecomposed litter seems to be a rather general phenomenon not restricted to few “allelopathic” plants. However, such inhibitory effect largely varied among litter types, and almost disappeared after 180 days of decomposition for all the tested litters, with the exception of two species (i.e. *Acanthus* and *Medicago*). Previous studies aimed at assessing litter allelopathic effects were criticized for using one or only a few sensitive target species of little ecological relevance. Here, by using 14 different bioassay plants belonging to different functional groups, we were able to generalize these findings and to highlight species-specific response differences, with annual plants far more sensitive than perennial and woody species. In addition, the magnitude of seedling root growth inhibition was inversely proportional to seed size, with small-seeded plants being more sensitive to litter chemical effects.

Our findings raise two main questions: why is undecomposed litter broadly inhibitory to root growth and why does this harmful effect lessen during decomposition? About the first issue, we have to consider that the initial phase of decomposition basically consists of plant tissue breakdown and subsequent release of cell contents. At this stage, the inhibitory effect showed a large variability among litter types, being high for materials from nitrogen fixing species such as *C*. *emerus* and *M*. *sativa*, but also for *P*. *abies*, *A*. *unedo*, *C*. *sativa*, and *A*.*mollis*, and low for other species (e.g. *C*. *sempervirens*, *P*. *nigra* and *F*. *sylvatica*). Root growth inhibition by undecomposed plant litter has been attributed to litter N immobilization^23^ or, alternatively, to the phytotoxic activity by allelopathic compounds^17^. The nutrient immobilization hypothesis sustains that litter with high C/N ratio, by reducing nitrogen relative availability, would limit root system development. However, such hypothesis is not supported by the present work, because litter N content and C/N ratio in our bioassays was unrelated to root proliferation for all target plant species. This indirectly suggests that other causal factors should be taken into account to explain the general inhibitory effects of undecomposed plant litter, although N limitation may still hold under specific ecological conditions, as in the case of litter with high C/N ratio in N poor soils^22, 54, 55^.

The alternative hypothesis states that plant litter can release, either directly or mediated by microbial activity, phytotoxic compounds such as phenols, aromatic rings, and tannins with harmful effects on root growth^17^. The use of ^13^C-CPMAS NMR allowed the depiction of biochemical differences among litter materials, thereby providing useful insights to explaining the variable effect on root growth. Two positive correlations were found between root growth of annuals and woody plants with the methoxyl and aromatic regions, respectively. Both regions, being based on lignin-related NMR signals^2, 56^, indicate that highly lignified plant materials have limited inhibitory effects on root proliferation. Moreover, our extensive correlative analysis applied along the whole ^13^C NMR spectrum provided a considerably improved definition of litter biochemical quality, compared with reference regions from literature^2, 57^, in relation to ecological functions. Specifically, we identified two restricted spectral intervals within the alkyl C, and the methoxyl C regions (3–24 ppm and 50–60 ppm, respectively), positively associated with root growth, and two other intervals within the *O*-alkyl C and di-*O*-alkyl C regions (67–80 ppm and 96–106 ppm, respectively) negatively correlated with root growth. These spectral signals correspond to specific molecular components of plant litter: the aliphatic chain in lignin and the acetate of some hemicelluloses, the methoxyl C of lignin, the C2, C3, and C5 of labile carbohydrates, and the C1 of cellulose^2, 41^.

Our correlative analysis also helps to explain the specific sensitivity to undecomposed litter of different plant functional groups (annuals≫ perennials ≥ woody). The five annual species showed a remarkably similar response to litter biochemical quality, with root growth being positively and negatively associated to methoxyl C and *O*-alkyl C, respectively. Proximate analysis confirmed evidence from NMR data, with root growth of annual plants, but not that of perennial and woody species, negatively associated to labile C. These results indicate that short-lived plants are far more inhibited, compared to long-lived species, by simple carbohydrates and other compounds that rapidly disappear during decomposition, such as condensed tannins^45, 58^. Root growth of short-lived plants was also positively related to specific spectral signals corresponding to different aromatic C types (122–126 ppm, 133–138 ppm and 151–153 ppm). The signals resonating at 151–153 ppm were previously attributed to N-substituted unsaturated carbons from heterocyclic aromatic amines, diagnostic of the N bases of nucleic acids^59^. These were previously associated with a species-specific effect on plant root growth, with inhibition and stimulation on conspecific and heterospecific species, respectively^31^.

The absence of sensitivity to different litter fractions in perennial and woody plants is yet to be clarified. In this regard, it is worth noting that some species (e.g. *Pinus*) showed a limited sensitivity to plant litter effects, as well as the absence of significant relationships between root growth and litter quality as defined by ^13^C NMR.

The higher sensitivity of short-lived and small-seeded plants to the chemical component of undecomposed litter is consistent with several field studies reporting the rapid disappearance of short-lived species in old fields, as a result of litter accumulation^12, 13, 48^. Considering the molecular and physiological basis of plant sensitivity to undecomposed litter, in relation to seed size and lifespan, the reserve effect hypothesis^60^ may provide a partial explanation. According to such hypothesis, small seeds contain too little reserve tissue to face environmental stresses such as litter phytotoxic effect. Previous studies related this negative effect of the litter layer to the light requirement and sensitivity to mechanical impediment of small-seeded plants^50^. In this context, we add a further possible mechanistic hypothesis, based on a direct negative effect of litter chemicals on seedling root growth of species with small seeds. At the field scale, most experiments deal with seedling emergence or establishment and not with the underlying processes^12, 13^. Therefore, future studies are required to reveal the relative contributions of mechanical impediment, light requirement and chemical interference by litter on the inhibition of small seeded, short-lived plant species.

### Litter preference by bacteria and fungi

In our experiments, about half of the tested fungi and bacteria thrived when exposed to the majority of undecomposed litter materials, with growth remarkably higher, not different, or slightly lower to that recorded on the controls over rich, standard microbiological substrates. However, a large response variability was found depending on litter type, with higher growth over materials from herbaceous, nitrogen-fixing (i.e. *Coronilla* and *Medicago*) and fast decomposing species (e.g. *Hedera*, *Salix*, and *Fraxinus*). A significant inhibition over litter from coniferous (i.e. *Cupressus*, *Picea*, and *Pinus*), slow decomposing deciduous trees (e.g. *Quercus*, *Fagus*), and perennial grasses (i.e. *Ampelodesmos* and *Festuca*) was recorded, but also in these cases all the bacteria and fungi were able to use these substrates for growth (Supplementary Fig. S1). In this regard, it is interesting to note the higher saprophytic capability of the selected fungi compared to bacteria. Opposite to higher plant response, a very steep decline of microbial growth was recorded over all litter decomposed for 180 days, compared to fresh materials, consistent for all bacterial and fungal species. These results confirm previous finding^40, 61^, reporting a high growth rate of 18 fungal species over several undecomposed leaf litter, followed by a rapid reduction of fungal development as litter decomposition proceeded. At first glance, these results could appear surprising, because several undecomposed litter used in this study are known to be rich in antifungal and antibacterial compounds. For instance, antimicrobial activity of pure molecules and/or leaf extracts obtained with organic solvents (e.g. butanol, ethyl acetate, methanol, etc.) has been reported for *A*. *mauritanicus* ^62^, *A*. *unedo* ^63^, *H*. *helix* ^64^, *P*. *nigra* ^65^, *R*. *pseudoacacia* ^66^ and *Q*. *ilex* ^67^. However, the use of organic solvents, compared to the water extracts used in our bioassays, allows a more complete extraction of organic compound from the starting leaf material, which is useful for phytochemical and toxicology assessments, but it also provides a less reliable reproduction of ecological processes in field conditions.

The dramatic decline of bacterial and fungal growth observed on aged litter, compared to undecomposed materials, can be explained by a progressive decrease in litter biochemical quality, with the substrate becoming less suitable to microbial exploitation. Such changes could be related to a decrease of easily degradable C sources, and to the accumulation of toxic and/or recalcitrant organic compounds. Results of proximate chemical analyses and ^13^C-CPMAS NMR highlight that both processes occur in ageing litter, with a sharp decrease of the labile C fraction and a relative increase of aromatic compounds^19, 40^. Surprisingly, proximate cellulose was unrelated to microbial growth, despite targeted fungi, and especially *Trichoderma harzianum*, are able to use it as a C source. Differently, ^13^C NMR data showed a positive association between signals within the di-*O* alkyl C region, diagnostic of the C1 of cellulose^41^, and fungal growth, although with species-specific differences. These contradictory results could be partially explained considering that a large cellulose proportion is entrapped with lignin, so that decomposition cannot proceed independent of lignin degradation^68^.

Microbial growth was also independent of litter N content. Previous studies reported a dual, contrasting effect of N on both litter decay^69, 70^ and fungal growth^40^, with high N content in litter promoting decomposition at the early stage (up to 30–40% of mass loss) and slowing it thereafter^41^. We suggest that high N content initially sustains a rapid microbial growth, while at later decay stages high N concentration could favor the formation of recalcitrant compounds with potential inhibitory effect^41^. However, the underlying microbiological and biochemical processes have not yet been clarified. In general terms, we found a pattern of association, between microbial growth and restricted ^13^C-CPMAS NMR spectral regions in litter, generally consistent among species, likely emerging from the balance between labile C sources availability and the occurrence of recalcitrant and/or toxic compounds. However, inter-specific response variability was higher for bacteria, suggesting that our approach could be more appropriate to address species level questions, and lower among fungi with the six tested species showing an exceptionally similar pattern. Such clear-cut behavior showed the same magnitude but an opposite direction to what was observed for higher plants, and for annual species in particular. Although our study design was not specifically aimed to compare microbes and annual plants coexisting in nature, our results suggest a complementary effect of litter on these two groups of organisms, whose occurrence in field conditions should be assessed. Along this line, saprotrophs would exploit fresh leaf materials being limited by aged and lignified litter, while plants would be inhibited by phytotoxic fresh litter with seed size- and lifespan-dependent magnitude, then thriving over aged substrates.

## Conclusions

Our multi-species bioassay approach revealed that plant litter has a species-specific effect on bacteria, fungi and higher plants. It is noteworthy that we found undecomposed plant litter mainly acts as a C source for saprophytic microbes, although microbial responses largely varied across litter types. In contrast, fresh litter hampers plant root growth, with severe inhibition observed for most plant species. An opposite response was found in bioassays with aged litter that largely inhibits microbial growth, but has neutral or stimulatory effects on root proliferation. The use of ^13^C-CPMAS NMR provides an improved definition of litter molecular quality, helping to explain the variable effects of plant litter on different microbial food web levels. ^13^C-CPMAS NMR revealed that restricted resonance intervals within the alkyl C, methoxyl C, *O*-alkyl C and di-*O*-alkyl C spectral regions are crucial for understanding litter effects, with plant root growth negatively affected by labile C, but positively associated with signals related to plant tissue lignifications. The opposite response was observed for bacteria and especially fungi to the corresponding ^13^C-CPMAS NMR signals. Despite limitations of species-specific bioassay methodology, we demonstrate that litter molecular quality can promote or inhibit functional species, and potentially control successional dynamics through time. These results emphasize the importance of simultaneously studying different microbial food web components in order to fully understand the effects of plant detritus on ecosystem structure and functionality.

## Materials and Methods

### Target bacteria, fungi and plants

Twenty-six target species were selected to assess substrate preference in bioassays (Supplementary Table S6; all species were obtained from collections at the Department of Agriculture, University of Naples Federico II). The species pool included six bacteria, six fungi with different functional traits and fourteen plants including five annuals, four perennial forbs and grasses and five evergreen or deciduous trees. Besides differing by life span and growth form, the selected plant species also showed a wide range of seed size (Supplementary Table S6).

### Plant litter types

Plant litter derives from a previous decomposition experiment focused on mass loss dynamics^47^, based on the litterbag method^41^. The litterbag experiment, based on eighteen plant species selected to represent a wide range of leaf litter quality, was carried out in controlled optimal conditions of temperature and water availability (further details in Supplementary Methods S1). The experiment produced 36 litter materials, either undecomposed (thereafter indicated as 0 days) or decomposed for 180 days. Biochemical quality of the 36 materials was previously reported^47^ and used as a reference dataset for plant residue biochemical quality in relation to bioassay results. Data include total C and N contents, labile C, proximate cellulose^51^ and spectral data from ^13^C-CPMAS NMR in solid state (for details see Supplementary Methods S1 and Bonanomi *et al*.^19^). Statistical analyses were done according to Supplementary Methods S5.

### Plant and microbe bioassays

For higher plants, “root proliferation” bioassays were conducted in root observation chambers^19, 71^. The aim of the bioassay was to assess the capability of seedling roots of the 14 target species to colonize the 36 different litter types, independent of the germination process. The experimental set up is fully reported in Supplementary Fig. S2 and in Supplementary Methods S2. Treatments, each with 10 replicates, included application of dry, powdered litter (separately for each of the 18 plant litter species at the 2 decomposition stages, for a total of 36 treatments) at a rate within the range observed in natural ecosystems considering the amount of litterfall and standing litter^72^, or distilled water as the control (Supplementary Methods S2). At the end of the experiment seedling root length was measured by digital photography and the total root length was measured by image analyser software (LUCIA; Laboratory Imaging Ltd. version 4.51).

Bioassays with bacteria and fungi aimed at assessing the effects of plant litter on target species growth in absence of inter-specific competition, thus allowing the evaluation of the relationships between litter biochemistry and microbial saprophytic growth. Treatments for bacteria were litter water extracts, obtained and applied as specified in Supplementary Methods S3, while Nutrient Broth, a common microbiological substrate, was used as control. Bacterial growth was spectrophotometrically measured at 6, 12, 24 and 48 hours of incubation after the inoculum (Supplementary Methods S3) using a Thermomax microtitre plate reader (Molecular Devices, Wokingham, UK). For data analysis, we used growth recorded after 48 hours and species response was expressed as percentage of the untreated control.

Treatment solutions for fungi were prepared as described in Supplementary Methods S4, in order to test the capability of fungi to use plant litter as the only source of nutrient, with Potato dextrose agar (PDA, Fluka), a common mycological substrate, used as the control. Seven days after the inoculum (Supplementary Methods S4), hyphal density and radial growth of each colony were assessed as described in Supplementary Methods S4. A fungal growth index was calculated as the product of the area of fungal colony, calculated from the measured colony radius, and hyphal density, following Tuitert *et al*.^73^.

Growth data from all bioassays were expressed as either percent of the untreated controls (i.e. control value = 100) or percent difference compared to the controls (i.e. control value = 0) and statistically analysed as described in Supplementary Methods S5

## Electronic supplementary material

Supplementary information
