# Supplementary material for: Litter chemistry explains contrasting feeding preferences of bacteria, fungi, and higher plants
Source: Sci Rep. 2017 Aug 23;7:9208. doi: 10.1038/s41598-017-09145-w (PMC5569010; doi:10.1038/s41598-017-09145-w)
Supplement: Supplementary file 1 — Supplementary information [file 41598_2017_9145_MOESM1_ESM.doc]

# Supplementary Information

**Litter chemistry explains contrasting feeding preferences of bacteria, fungi and higher plants**

Giuliano Bonanomi^1*^, Gaspare Cesarano^1^, Nadia Lombardi^1^, Riccardo Motti^1^, Felice Scala^1^, Stefano Mazzoleni^1^, Guido Incerti^2^

*^1^* *Dipartimento di Agraria, University of Naples Federico II, via Università 100, 80055 Portici (NA), Italy*

*^2^* *Department of Agri-Food, Animal and Environmental Sciences, University of Udine, via delle Scienze 206, 33100 Udine, Italy*

**Corresponding author:*

E-mail: giuliano.bonanomi@unina.it – Tel. +39 081 2539015

Submitted to: *Scientific Reports*

Number of text pages (including this cover page): 13

Number of tables: 6

Number of figures: 2

# Supplementary Methods S1 - Plant litter types

Plant litter derives from a previous litterbag decomposition experiment focused on mass loss dynamics^47^, hereafter briefly summarized. Eighteen different plant species, including two perennial grasses (*Ampelodesmos mauritanicus*, *Festuca drymeia*), two perennial forbs (*Acanthus mollis* and *Medicago sativa*) two evergreen shrubs (*Arbutus unedo*, *Coronilla emerus*), one vine (*Hedera helix*), four evergreen trees (*Cupressus sempervirens, Picea abies*, *Pinus halepensis, Quercus ilex*), and seven deciduous trees (*Alnus glutinosa,* *Castanea sativa*, *Fagus sylvatica*, *Fraxinus ornus*, *Populus nigra*, *Robinia pseudoacacia*, and *Salix alba*) were selected as representing a wide range of leaf litter quality. Decomposition experiments were carried out in microcosms according to the litterbag method^41^. Large (20 x 20 cm^2^) terylene litterbags (mesh size 2 x 2 mm^2^) were filled with 6 g of undecomposed, dry leaf litter and placed inside trays (100 x 100 x 30 cm^3^). Microcosms were kept in a growth chamber under controlled temperature (18±2°C night and 24±2°C day) and water (watered every seven days to field capacity with distilled water) conditions. Litterbags (8 replicates) were harvested after 180 days of decomposition. Bags were dried in the laboratory (40°C until constant weight was achieved) and the remaining material weighed. In this way, 36 organic materials (18 species at two sampling dates) with different ages were produced: fresh undecomposed litter (thereafter indicated as 0 days) and litter decomposed for 180 days.

Biochemical quality of the 36 materials was previously reported^47^. These data were used as a reference dataset of plant residues biochemical quality and related to bioassays results. Briefly, the 36 materials were characterized for total C and N contents, labile C and proximate cellulose^51^ as well as spectral data from ^13^C-CPMAS NMR in solid state (for details see Bonanomi et al.^19^). Selection of spectral regions and identification of C-types were performed according to previous studies^2,66,80^. The following seven regions and C types were considered: 0-45 p.p.m = alkyl C; 46-60 ppm = methoxyl C; 61-90 ppm = *O*-alkyl C; 91-110 ppm = di-*O*-alkyl C; 111-140 ppm = H- and C- substituted aromatic C; 141-160 ppm O-substituted aromatic C (phenolic and O-aryl C); 161-190 ppm carboxyl C. Two indicators of organic matter decomposition progress were calculated: (i) the alkyl C / *O*-alkyl C ratio; (ii) the *O*-alkyl C / methoxyl and N-alkyl C ratio (thereafter indicated as CC/MC)^65,66^.

# Supplementary Methods S2 - Plant bioassays

For higher plants, “root proliferation” bioassays were conducted in root observation chambers^19,81^. Aim of the bioassay was to assess the capability of seedling roots of the 14 target species to colonize the 36 different litter types, independent of the germination process. A 2 cm wide sterile filter paper strip wetted with distilled water was placed immediately above a wider (5 cm) strip in square Petri dishes (size 12 x 12 x 1.5 cm). The two strips were separated by 5 mm free space. Seeds (5 for each dish) were placed over the 2 cm strip, whereas litter was applied on the bottom 5 cm wide strip. This set up was used to ensure that seeds could germinate only over the filter paper wetted with distilled water, thus preventing litter interference with the germination process. Not germinated seeds where replaced to ensure that the same number of growing seedlings were monitored in each dish. After seed germination, seedling rootlet started to proliferate entering in contact with the litter material. The experimental set up is illustrated in Supplementary Fig. S2. Petri dishes were oriented with a 45° slope on a horizontal surface so that positive geotropism would allow rootlet growth downwards along the plate. Plates were covered with opaque sheets when roots were not under observation. The experimental design included two treatments: 1) control with sterile distilled water; 2) litter addition on the 5 cm wide paper strip. Dry, powdered litter (separately for each of the 18 plant species at the 2 decomposition stages, for a total of 36 treatments) was applied at 0.2 g / dish. Experimental values of litter addition were within the range observed in natural ecosystems considering the amount of litterfall and standing litter^82^. Each treatment was replicated 10 times. A total of 5,180 experimental units were prepared (14 target species × 36 litter types plus untreated control × 10 replicates). Petri dishes were arranged in a growing room according to a totally randomized design. The growing room was kept under controlled temperature (16±2°C night and 22±2°C day) and relative humidity (55%) conditions. The experiment duration varied among target species depending on root growth rate in the control treatment (Table S6). At the end of the experiment seedling root length was measured by digital photography and the total root length was measured by image analyser software (LUCIA; Laboratory Imaging Ltd. version 4.51).

# Supplementary Methods S3 - Bacteria bioassays

Bioassays with bacteria and fungi aimed to assess the effects of plant litter on target species growth in absence of inter-specific competition. This approach specifically allows evaluating the relationships between litter biochemistry and microbial saprophytic growth. Treatments for bacteria were litter water extracts, obtained by mixing dried litter material in a beaker with distilled water at 5% of dry weight (50 g l^-1^) and shaken for 5 h. The aqueous suspensions were then centrifuged (2395 *g* for 10 min), sterilized (micro filtration with 0.22 μm pore filter) and stored at -20°C.

After checking sterility by plating on nutrient agar broth (NA, Oxoid) and potato dextrose agar (PDA, Fluka), litter extracts (36 types, 100 µl) were applied in 96-well plates and incubated at 28 °C with ten replicates. Nutrient Broth, a common microbiological substrate, was used as control. The inoculum of bacterial strains was 10 µL of physiological solution (NaCl 0.9% w/v) with concentration of 10^3^ cells mL^-1^. Bacterial growth was spectrophotometrically measured (λ= 530 nm) after 6, 12, 24 and 48 hours of incubation using a Thermomax microtitre plate reader (Molecular Devices, Wokingham, UK). For data analysis, we used growth recorded after 48 hours and species response was expressed as percentage of the untreated control.

# Supplementary Methods S4 - Fungi bioassays

Treatment solutions for fungi were prepared by mixing each of the 36 sterile litter extracts with water agar, a substrate composed only by agar without added nutrients (WA, Oxoid), in order to test the fungi capability to use plant litter as the only source of nutrient. Potato dextrose agar (PDA, Fluka), a common mycological substrate, was used as the control. Ten millilitres of the treatment solution were placed in a 9 cm Petri dish, and each treatment was replicated ten times. Fungal inoculum was prepared from colonies growing on WA at 24°C. After seven days of culture, a 4 mm diameter plug was collected from the edge of the growing colony and placed in the centre of a Petri dish. After 72 h, hyphal density and radial growth of each colony were measured on five randomly chosen points. Hyphal density was measured by counting the number of hyphae crossing a 1 mm line at 250× amplification under binocular microscope. A fungal growth index was calculated as the product of the area of fungal colony, calculated from the measured colony radius, and hyphal density, following Tuitert et al.^83^.

# Supplementary Methods S5 – Data analysis

For statistical analysis of the bioassay results, data were submitted to Generalized Linear Mixed Modeling (GLMM), considering target species response expressed as percentage of the untreated control as the dependent variable. To assess differences of litter effects among groups of target organisms, a GLMM on data pooled for all bioassays was tested, including main and second order interactive effects of the group of organisms (3 levels: either plants, fungi or bacteria), litter species (random effect with 18 levels) and age (2 levels, either fresh or decomposed). GLMMs were also separately tested for plants, bacteria and fungi, including the effect of target species (treated as a random effect with 14, 6 and 6 levels for plants, bacteria and fungi, respectively) in addition to the random and fixed effects of litter species and age. Finally, limited to plants, a different GLMM was also tested, with the random effect of target species replaced by a fixed effect with 3 levels, according to plant functional types (annual, perennial or woody), in order to test response differences among target plant species corresponding to functional differences. Pairwise differences were tested using Tukey's HSD post-hoc test. Statistical significance was evaluated in all cases at *P* < 0.05.

The relationship between bioassay performances and litter chemistry parameters (i.e. N content, labile C, cellulose content, C/N ratio) was assessed by Pearson's correlation analysis. Then, a data matrix of ^13^C-CPMAS NMR spectral signals recorded in litter samples was submitted to a Principal Component Analysis (PCA). Litter chemical parameters and bioassay results on each material, averaged for plant functional groups, fungi and bacteria, were included in the PCA and treated as supplementary variables according to Legendre and Legendre^84^. In addition to data mining, Pearson's correlation analysis was carried out to identify parameters and regions of the ^13^C-CPMAS NMR spectra significantly associated to target species response in the bioassays. This was done by testing ^13^C-CPMAS NMR spectral regions selected from reference literature^2,65,66^ and by testing all consecutive signals along the spectrum for significant linear correlation with target species growth. Correlation was also tested between plant seed weight, expressed on a logarithmic scale, and seedling root growth on litter materials. Statistical significance in correlation analyses was set after controlling for multiple comparison, according to the FDR (False Discovery Rate) approach^85^.

# References in Supplementary Methods

Reference numbers in Supplementary methods refer to main text.

## **Supplementary Table S1.** Correlation (Pearson's *r*) between ^13^C CAMS NMR signals recorded in 36 litter types and the growths of 6 bacteria incubated with the same litter materials. Bacterial species are as follows: *Bacillus subtilis* (Bac)*, Erwinia carotovora* (Erw)*, Escherichia coli* (Esc)*, Lysobacter gummosus* (Lys)*, Pseusomonas fluorescens* (Pse) and *Rhizobium radiobacter* (Rhi). Statistically significant (*p* < 0.01 after controlling for multiple comparison according to Benjamini and Hochberg^85^) and marginally significant (0.01 < *p* < 0.05) correlation values are highlighted in italic and bold, respectively.

| **^13^C NMR**  **(ppm)** | **Correlation with bacterial growth** | | | | | |  | **^13^C NMR**  **(ppm)** | **Correlation with bacterial growth** | | | | | |
| --- | --- | --- | --- | --- | --- | --- | --- | --- | --- | --- | --- | --- | --- | --- |
|  | **Bac** | **Erw** | **Esc** | **Lys** | **Pse** | **Rhi** |  |  | **Bac** | **Erw** | **Esc** | **Lys** | **Pse** | **Rhi** |
| *190-155 | - | - | - | - | - | - |  | 78 | 0.31 | 0.14 | *0.38* | 0.31 | **0.44** | 0.04 |
| 154 | -0.04 | *-0.35* | -0.26 | -0.04 | -0.3 | -0.14 |  | 77 | 0.29 | 0.13 | *0.36* | 0.27 | **0.44** | 0.06 |
| 153 | -0.17 | **-0.45** | **-0.43** | -0.23 | **-0.45** | -0.12 |  | 76 | 0.3 | 0.14 | *0.36* | 0.25 | **0.43** | 0.06 |
| 152 | -0.27 | **-0.46** | **-0.47** | -0.29 | **-0.45** | -0.06 |  | 75 | 0.32 | 0.18 | *0.38* | 0.25 | **0.44** | 0.06 |
| 151 | -0.33 | *-0.39* | **-0.45** | -0.25 | *-0.42* | -0.02 |  | 74 | *0.35* | 0.26 | **0.44** | 0.28 | **0.47** | 0.05 |
| 150 | -0.27 | *-0.36* | *-0.37* | -0.16 | *-0.35* | 0.01 |  | 73 | *0.41* | *0.33* | **0.49** | 0.3 | **0.48** | 0.02 |
| 149 | -0.2 | *-0.35* | -0.33 | -0.09 | *-0.36* | -0.02 |  | 72 | **0.47** | *0.4* | **0.52** | 0.31 | **0.44** | -0.04 |
| 148 | -0.06 | *-0.35* | -0.27 | 0 | *-0.34* | -0.16 |  | 71 | **0.52** | **0.47** | **0.57** | *0.35* | *0.41* | -0.05 |
| 147 | 0.16 | -0.13 | 0.03 | 0.25 | -0.1 | -0.13 |  | 70 | **0.54** | **0.5** | **0.61** | *0.38* | **0.43** | -0.02 |
| 146 | *0.33* | -0.04 | 0.22 | **0.45** | 0.05 | -0.13 |  | 69 | **0.53** | **0.48** | **0.61** | *0.39* | **0.45** | -0.02 |
| 145 | *0.38* | 0.01 | 0.29 | **0.5** | 0.1 | -0.13 |  | 68 | **0.47** | *0.41* | **0.54** | *0.34* | *0.4* | 0.03 |
| 144 | *0.33* | 0 | 0.25 | **0.49** | 0.07 | -0.16 |  | 67 | *0.33* | 0.17 | *0.35* | 0.2 | *0.34* | 0.06 |
| 143 | 0.21 | -0.06 | 0.12 | *0.39* | -0.03 | -0.15 |  | *66-61 | - | - | - | - | - | - |
| *142-139 | - | - | - | - | - | - |  | 60 | *-0.34* | -0.23 | **-0.43** | *-0.39* | *-0.42* | -0.02 |
| 138 | *-0.34* | -0.32 | *-0.41* | -0.09 | **-0.43** | -0.08 |  | 59 | *-0.42* | -0.31 | **-0.51** | **-0.43** | **-0.49** | 0 |
| 137 | *-0.35* | *-0.38* | **-0.46** | -0.16 | *-0.42* | -0.13 |  | 58 | **-0.47** | *-0.34* | **-0.56** | **-0.46** | **-0.51** | 0.02 |
| 136 | *-0.42* | **-0.43** | **-0.55** | -0.33 | **-0.47** | -0.06 |  | 57 | **-0.46** | *-0.34* | **-0.54** | **-0.43** | **-0.5** | 0.03 |
| 135 | -0.3 | **-0.48** | **-0.53** | -0.32 | *-0.42* | -0.17 |  | 56 | **-0.45** | -0.3 | **-0.53** | **-0.44** | **-0.54** | 0.01 |
| 134 | -0.25 | **-0.5** | **-0.55** | *-0.35* | **-0.51** | -0.24 |  | 55 | *-0.4* | -0.25 | **-0.5** | **-0.44** | **-0.51** | -0.04 |
| 133 | -0.12 | **-0.49** | **-0.46** | -0.25 | **-0.5** | -0.2 |  | 54 | *-0.35* | -0.15 | *-0.42* | *-0.41* | **-0.45** | -0.03 |
| 132 | -0.03 | *-0.42* | *-0.42* | -0.25 | **-0.48** | *-0.36* |  | 53 | *-0.39* | -0.12 | *-0.42* | *-0.36* | **-0.43** | -0.07 |
| 131 | -0.05 | *-0.35* | *-0.38* | -0.26 | *-0.39* | *-0.34* |  | 52 | *-0.37* | -0.13 | *-0.42* | *-0.35* | *-0.4* | -0.12 |
| *130-129 | - | - | - | - | - | - |  | 51 | *-0.35* | -0.14 | *-0.4* | *-0.33* | *-0.4* | -0.08 |
| 128 | -0.1 | -0.26 | -0.28 | *-0.38* | -0.21 | -0.02 |  | 50 | *-0.35* | -0.12 | *-0.37* | -0.33 | *-0.36* | -0.05 |
| 127 | -0.18 | -0.28 | *-0.35* | *-0.37* | -0.33 | -0.03 |  | *49-25 | - | - | - | - | - | - |
| 126 | -0.25 | *-0.38* | **-0.44** | *-0.35* | *-0.4* | -0.06 |  | 24 | *-0.34* | -0.09 | -0.29 | -0.24 | -0.26 | 0.04 |
| 125 | *-0.33* | **-0.47** | **-0.56** | *-0.38* | **-0.5** | -0.13 |  | 23 | *-0.38* | -0.14 | *-0.36* | -0.27 | *-0.34* | 0.03 |
| 124 | *-0.33* | **-0.48** | **-0.57** | *-0.37* | **-0.55** | -0.11 |  | 22 | *-0.39* | -0.14 | *-0.38* | -0.27 | *-0.39* | 0.02 |
| 123 | -0.2 | *-0.4* | *-0.42* | -0.22 | **-0.45** | -0.14 |  | 21 | *-0.38* | -0.12 | *-0.36* | -0.24 | *-0.41* | 0.05 |
| 122 | -0.19 | *-0.39* | *-0.38* | -0.12 | *-0.41* | -0.1 |  | 20 | *-0.37* | -0.11 | -0.33 | -0.2 | -0.33 | 0.01 |
| 121 | -0.07 | *-0.39* | -0.27 | -0.02 | -0.32 | -0.09 |  | 19 | *-0.38* | -0.08 | -0.29 | -0.18 | -0.29 | 0.07 |
| *120-112 | - | - | - | - | - | - |  | 18 | *-0.4* | -0.08 | -0.29 | -0.17 | -0.29 | 0.12 |
| 111 | -0.03 | *-0.34* | -0.17 | 0.18 | -0.15 | -0.19 |  | 17 | *-0.4* | -0.08 | -0.29 | -0.16 | -0.29 | 0.12 |
| *110-107 | - | - | - | - | - | - |  | 16 | *-0.41* | -0.14 | *-0.36* | -0.25 | *-0.34* | 0.07 |
| 106 | 0.21 | 0.01 | 0.23 | 0.16 | *0.33* | 0.07 |  | 15 | *-0.41* | -0.17 | *-0.37* | -0.28 | *-0.37* | 0.09 |
| 105 | 0.21 | 0.13 | -0.02 | 0.07 | 0.29 | 0.20 |  | 14 | **-0.44** | -0.18 | *-0.4* | -0.3 | *-0.39* | 0.1 |
| 104 | 0.19 | 0.11 | -0.02 | 0.08 | 0.28 | 0.18 |  | 13 | **-0.44** | -0.18 | *-0.41* | -0.3 | *-0.39* | 0.05 |
| 103 | 0.25 | 0.07 | 0.28 | 0.16 | *0.35* | 0.09 |  | 12 | *-0.41* | -0.21 | *-0.42* | -0.32 | *-0.4* | 0.02 |
| 102 | *0.38* | 0.14 | *0.34* | 0.2 | 0.32 | -0.01 |  | 11 | *-0.41* | -0.22 | **-0.44** | -0.32 | *-0.42* | 0.01 |
| 101 | **0.45** | 0.18 | *0.38* | 0.24 | 0.31 | -0.05 |  | 10 | *-0.41* | -0.2 | *-0.42* | *-0.33* | *-0.38* | 0.03 |
| 100 | **0.48** | 0.12 | *0.33* | 0.22 | 0.22 | -0.15 |  | 9 | *-0.36* | -0.16 | *-0.35* | -0.25 | *-0.34* | 0.03 |
| 99 | **0.54** | 0.12 | *0.34* | 0.21 | 0.2 | -0.22 |  | 8 | *-0.37* | -0.16 | *-0.35* | -0.23 | *-0.34* | 0.05 |
| 98 | **0.52** | 0.17 | *0.35* | 0.29 | 0.19 | -0.25 |  | 7 | *-0.36* | -0.11 | -0.32 | -0.23 | *-0.34* | 0.06 |
| 97 | **0.46** | 0.18 | *0.33* | 0.32 | 0.2 | -0.26 |  | 6 | *-0.34* | -0.17 | *-0.34* | -0.18 | -0.32 | -0.03 |
| 96 | *0.42* | 0.18 | *0.34* | *0.35* | 0.26 | -0.26 |  | 5 | *-0.38* | -0.22 | *-0.41* | -0.28 | *-0.38* | -0.03 |
| *95-82 | - | - | - | - | - | - |  | 4 | *-0.36* | -0.26 | *-0.42* | *-0.34* | *-0.36* | 0.02 |
| 81 | 0.25 | 0.09 | 0.29 | 0.23 | *0.35* | 0.05 |  | 3 | -0.33 | -0.22 | *-0.36* | -0.26 | *-0.34* | 0.02 |
| 80 | 0.29 | 0.13 | *0.36* | 0.3 | *0.39* | 0.07 |  | 2 | -0.31 | -0.22 | *-0.37* | -0.21 | *-0.38* | -0.06 |
| 79 | *0.34* | 0.13 | *0.38* | 0.33 | *0.42* | 0.05 |  | 1 | -0.31 | -0.24 | *-0.39* | -0.23 | *-0.39* | -0.09 |

*: Ranges of consecutive spectral signals for which non-significant correlations were observed for all target species are omitted.

## **Supplementary Table S2.** Results as in Table S2, but for 6 fungal species: *Aspergillus niger* (Asp), *Botrytis cinerea* (Bot), *Ganoderma lucidum* (Gan), *Mucor* sp. (Muc), *Trichoderma* *harzianum* (Tri), *Umbelopsis ramanniana* (Umb).

| **^13^C NMR**  **(ppm)** | **Correlation with fungal growth** | | | | | |  | **^13^C NMR**  **(ppm)** | **Correlation with fungal growth** | | | | | |
| --- | --- | --- | --- | --- | --- | --- | --- | --- | --- | --- | --- | --- | --- | --- |
|  | **Asp** | **Bot** | **Gan** | **Muc** | **Tri** | **Umb** |  |  | **Asp** | **Bot** | **Gan** | **Muc** | **Tri** | **Umb** |
| *190-154 | - | - | - | - | - | - |  | 71 | **0.64** | **0.56** | **0.56** | **0.59** | **0.61** | **0.63** |
| 153 | *-0.41* | *-0.35* | -0.33 | **-0.44** | *-0.40* | **-0.43** |  | 70 | **0.62** | **0.63** | **0.55** | **0.58** | **0.60** | **0.60** |
| 152 | **-0.47** | *-0.42* | *-0.39* | **-0.49** | **-0.45** | **-0.47** |  | 69 | **0.62** | **0.65** | **0.56** | **0.56** | **0.58** | **0.59** |
| 151 | **-0.48** | *-0.40* | *-0.41* | **-0.46** | **-0.44** | **-0.45** |  | 68 | **0.56** | **0.58** | **0.51** | **0.47** | **0.50** | **0.51** |
| 150 | *-0.36* | -0.29 | -0.31 | *-0.41* | *-0.36* | *-0.37* |  | 67 | **0.48** | *0.40* | **0.47** | 0.28 | *0.39* | *0.39* |
| *149-147 | - | - | - | - | - | - |  | 66 | *0.34* | 0.16 | *0.37* | 0.16 | 0.25 | 0.27 |
| 146 | 0.29 | *0.34* | 0.17 | 0.12 | 0.05 | 0.14 |  | 65 | 0.27 | 0.09 | *0.33* | 0.11 | 0.15 | 0.21 |
| 145 | 0.33 | **0.43** | 0.25 | 0.17 | 0.08 | 0.17 |  | *64-62 | - | - | - | - | - | - |
| 144 | 0.32 | *0.39* | 0.25 | 0.17 | 0.07 | 0.16 |  | 61 | *-0.38* | -0.29 | -0.30 | -0.28 | -0.17 | -0.28 |
| *143-140 | - | - | - | - | - | - |  | 60 | **-0.53** | *-0.42* | **-0.49** | *-0.39* | -0.31 | *-0.41* |
| 139 | -0.29 | -0.29 | *-0.37* | -0.28 | -0.28 | -0.29 |  | 59 | **-0.60** | **-0.52** | **-0.55** | **-0.48** | *-0.38* | **-0.50** |
| 138 | **-0.48** | *-0.40* | **-0.48** | **-0.42** | *-0.39* | **-0.44** |  | 58 | **-0.65** | **-0.57** | **-0.59** | **-0.53** | *-0.42* | **-0.56** |
| 137 | **-0.45** | *-0.34* | *-0.38* | **-0.43** | *-0.33* | *-0.42* |  | 57 | **-0.68** | **-0.56** | **-0.61** | **-0.55** | **-0.46** | **-0.58** |
| 136 | **-0.51** | **-0.46** | *-0.41* | **-0.49** | *-0.35* | **-0.47** |  | 56 | **-0.66** | **-0.55** | **-0.58** | **-0.51** | *-0.41* | **-0.55** |
| 135 | *-0.41* | *-0.38* | -0.28 | **-0.43** | -0.30 | *-0.40* |  | 55 | **-0.60** | **-0.50** | **-0.52** | *-0.42* | *-0.35* | **-0.45** |
| 134 | **-0.46** | *-0.40* | *-0.34* | *-0.42* | *-0.34* | **-0.44** |  | 54 | **-0.51** | **-0.44** | **-0.47** | -0.32 | -0.26 | *-0.34* |
| 133 | *-0.42* | *-0.34* | -0.32 | **-0.43** | *-0.41* | **-0.45** |  | 53 | **-0.52** | **-0.43** | **-0.51** | -0.31 | -0.28 | *-0.35* |
| *132-128 | - | - | - | - | - | - |  | 52 | **-0.53** | *-0.41* | **-0.51** | -0.32 | -0.31 | *-0.37* |
| 127 | *-0.39* | -0.28 | -0.24 | -0.33 | -0.30 | -0.30 |  | 51 | **-0.51** | *-0.41* | **-0.51** | -0.32 | -0.33 | *-0.37* |
| 126 | **-0.46** | -0.32 | *-0.36* | **-0.43** | *-0.40* | **-0.44** |  | 50 | **-0.52** | *-0.38* | **-0.54** | *-0.35* | *-0.35* | *-0.40* |
| 125 | **-0.51** | *-0.39* | *-0.38* | **-0.51** | *-0.39* | **-0.50** |  | 49 | **-0.43** | -0.26 | **-0.43** | -0.28 | -0.30 | -0.29 |
| 124 | **-0.53** | **-0.46** | *-0.42* | **-0.49** | *-0.41* | **-0.52** |  | 48 | *-0.38* | -0.23 | *-0.39* | -0.24 | -0.21 | -0.25 |
| 123 | *-0.39* | *-0.40* | *-0.33* | *-0.41* | -0.32 | *-0.41* |  | 47 | *-0.40* | -0.25 | *-0.39* | -0.26 | -0.18 | -0.27 |
| 122 | *-0.42* | -0.31 | *-0.40* | **-0.43** | **-0.43** | **-0.47** |  | 46 | *-0.40* | -0.24 | *-0.39* | -0.25 | -0.20 | -0.29 |
| 121 | -0.29 | -0.16 | -0.28 | *-0.34* | *-0.41* | *-0.39* |  | 45 | **-0.44** | -0.24 | **-0.47** | -0.27 | -0.28 | *-0.34* |
| 120 | -0.24 | -0.10 | -0.20 | -0.22 | *-0.38* | -0.32 |  | 44 | *-0.41* | -0.18 | **-0.46** | -0.23 | -0.26 | -0.32 |
| *119-109 | - | - | - | - | - | - |  | 43 | *-0.34* | -0.10 | *-0.38* | -0.17 | -0.18 | -0.24 |
| 108 | *0.34* | 0.10 | 0.30 | 0.11 | 0.12 | 0.19 |  | *42-31 | - | - | - | - | - | - |
| 107 | *0.38* | 0.15 | *0.38* | 0.15 | 0.18 | 0.25 |  | 30 | *-0.34* | -0.17 | *-0.35* | -0.21 | -0.22 | -0.28 |
| 106 | *0.41* | 0.19 | **0.43** | 0.20 | 0.22 | 0.31 |  | 29 | *-0.40* | -0.22 | *-0.41* | -0.27 | -0.28 | *-0.34* |
| 105 | *0.35* | 0.16 | *0.39* | 0.16 | 0.18 | 0.25 |  | 28 | *-0.41* | -0.25 | **-0.42** | -0.29 | -0.29 | *-0.35* |
| 104 | 0.29 | 0.17 | *0.34* | 0.14 | 0.13 | 0.20 |  | 27 | *-0.39* | -0.24 | *-0.39* | -0.26 | -0.24 | -0.31 |
| 103 | 0.32 | 0.25 | *0.37* | 0.22 | 0.18 | 0.24 |  | 26 | *-0.37* | -0.23 | *-0.37* | -0.22 | -0.20 | -0.28 |
| 102 | *0.40* | *0.37* | **0.44** | 0.31 | 0.30 | *0.34* |  | 25 | *-0.42* | -0.25 | *-0.41* | -0.26 | -0.24 | -0.33 |
| 101 | **0.47** | **0.45** | **0.49** | *0.34* | *0.37* | *0.39* |  | 24 | **-0.49** | -0.30 | **-0.48** | *-0.34* | -0.32 | *-0.40* |
| 100 | *0.41* | *0.41* | **0.42** | 0.30 | 0.31 | 0.33 |  | 23 | **-0.53** | *-0.37* | **-0.49** | *-0.38* | *-0.36* | **-0.43** |
| 99 | **0.44** | **0.43** | **0.47** | *0.37* | *0.34* | *0.38* |  | 22 | **-0.52** | **-0.43** | **-0.47** | *-0.36* | *-0.35* | *-0.42* |
| 98 | *0.42* | **0.43** | *0.42* | *0.38* | 0.28 | *0.35* |  | 21 | **-0.53** | *-0.41* | **-0.50** | *-0.36* | *-0.36* | **-0.43** |
| 97 | *0.38* | *0.41* | 0.32 | *0.38* | 0.22 | 0.31 |  | 20 | **-0.51** | -0.32 | **-0.52** | *-0.36* | *-0.38* | *-0.42* |
| 96 | **0.48** | *0.42* | *0.40* | *0.38* | 0.30 | *0.38* |  | 19 | **-0.51** | *-0.33* | **-0.55** | *-0.38* | *-0.40* | **-0.43** |
| 95 | *0.38* | 0.30 | *0.34* | 0.32 | 0.22 | 0.33 |  | 18 | **-0.51** | *-0.34* | **-0.56** | *-0.39* | *-0.42* | **-0.45** |
| 94 | *0.35* | 0.30 | 0.30 | 0.29 | 0.16 | 0.30 |  | 17 | **-0.49** | *-0.35* | **-0.54** | *-0.37* | *-0.42* | **-0.43** |
| 93 | *0.35* | 0.16 | 0.27 | 0.32 | 0.20 | *0.34* |  | 16 | **-0.53** | *-0.40* | **-0.53** | *-0.41* | *-0.41* | **-0.47** |
| *92-87 | - | - | - | - | - | - |  | 15 | **-0.54** | *-0.39* | **-0.50** | **-0.43** | *-0.39* | **-0.48** |
| 86 | *0.35* | 0.10 | *0.33* | 0.19 | 0.18 | 0.28 |  | 14 | **-0.55** | *-0.42* | **-0.52** | **-0.45** | *-0.41* | **-0.50** |
| 85 | *0.39* | 0.14 | *0.38* | 0.23 | 0.21 | 0.31 |  | 13 | **-0.54** | *-0.42* | **-0.50** | *-0.41* | *-0.40* | **-0.48** |
| 84 | *0.39* | 0.16 | *0.41* | 0.24 | 0.23 | 0.32 |  | 12 | **-0.55** | *-0.42* | **-0.49** | *-0.41* | *-0.41* | **-0.47** |
| 83 | *0.40* | 0.19 | *0.39* | 0.25 | 0.22 | 0.32 |  | 11 | **-0.55** | **-0.43** | **-0.49** | *-0.41* | *-0.41* | **-0.48** |
| 82 | **0.44** | 0.24 | *0.41* | 0.28 | 0.25 | *0.35* |  | 10 | **-0.54** | *-0.41* | **-0.49** | *-0.41* | *-0.40* | **-0.47** |
| 81 | **0.48** | 0.29 | **0.44** | 0.31 | 0.26 | *0.37* |  | 9 | **-0.45** | *-0.36* | *-0.42* | *-0.34* | *-0.34* | *-0.39* |
| 80 | **0.53** | *0.35* | **0.48** | *0.35* | 0.28 | *0.41* |  | 8 | **-0.46** | *-0.39* | **-0.48** | *-0.36* | *-0.36* | *-0.42* |
| 79 | **0.56** | *0.38* | **0.50** | *0.36* | 0.30 | **0.43** |  | 7 | **-0.44** | *-0.35* | **-0.45** | -0.32 | -0.33 | *-0.38* |
| 78 | **0.56** | *0.37* | **0.52** | *0.36* | *0.33* | **0.44** |  | 6 | **-0.43** | *-0.35* | *-0.42* | *-0.34* | *-0.38* | *-0.38* |
| 77 | **0.54** | *0.34* | **0.52** | *0.35* | *0.33* | **0.43** |  | 5 | **-0.47** | *-0.37* | *-0.42* | *-0.40* | *-0.34* | *-0.42* |
| 76 | **0.53** | 0.33 | **0.53** | *0.35* | *0.34* | **0.43** |  | 4 | **-0.51** | *-0.37* | **-0.45** | **-0.44** | *-0.41* | **-0.45** |
| 75 | **0.55** | *0.35* | **0.55** | *0.39* | *0.38* | **0.47** |  | 3 | **-0.47** | *-0.38* | **-0.44** | *-0.38* | *-0.39* | *-0.41* |
| 74 | **0.61** | *0.39* | **0.59** | **0.46** | **0.44** | **0.54** |  | 2 | *-0.38* | *-0.35* | *-0.34* | -0.32 | -0.30 | -0.32 |
| 73 | **0.66** | **0.44** | **0.63** | **0.52** | **0.52** | **0.60** |  | 1 | *-0.41* | *-0.35* | *-0.38* | *-0.35* | *-0.34* | *-0.35* |
| 72 | **0.66** | **0.49** | **0.61** | **0.56** | **0.58** | **0.63** |  |  |  |  |  |  |  |  |

*: Ranges of consecutive spectral signals for which non-significant correlations were observed for all target species are omitted.

## **Supplementary Table S3.** Results as in Tables S2-S3, but for 5 annual plants: *Arabidopsis thaliana* (Ara), *Lepidium sativum* (Lep), *Lycopersicon esculentum* (Lyc), *Trifolim pratense* (Trf), and *Triticum durum* (Tdu).

| **^13^C NMR**  **(ppm)** | **Correlation with root growth** | | | | |  | **^13^C NMR**  **(ppm)** | **Correlation with root growth** | | | | |
| --- | --- | --- | --- | --- | --- | --- | --- | --- | --- | --- | --- | --- |
|  | **Ara** | **Lep** | **Lyc** | **Trf** | **Tdu** |  |  | **Ara** | **Lep** | **Lyc** | **Trf** | **Tdu** |
| *190-186 | - | - | - | - | - |  | 72 | **-0.53** | **-0.56** | **-0.56** | **-0.62** | **-0.68** |
| 185 | 0.27 | 0.23 | *0.37* | 0.25 | 0.26 |  | 71 | *-0.42* | **-0.47** | **-0.52** | **-0.62** | **-0.66** |
| *184-173 | - | - | - | - | - |  | 70 | -0.31 | *-0.41* | **-0.48** | **-0.62** | **-0.63** |
| 172 | *0.41* | 0.26 | 0.29 | 0.23 | 0.32 |  | 69 | -0.26 | *-0.40* | **-0.45** | **-0.61** | **-0.61** |
| 171 | *0.42* | 0.25 | 0.30 | 0.25 | *0.33* |  | 68 | -0.19 | -0.32 | *-0.38* | **-0.55** | **-0.56** |
| *170-154 | - | - | - | - | - |  | 67 | -0.31 | -0.26 | *-0.35* | *-0.41* | **-0.51** |
| 153 | *0.36* | 0.33 | 0.33 | 0.29 | *0.39* |  | 66 | *-0.36* | -0.21 | -0.24 | -0.24 | *-0.36* |
| 152 | *0.42* | *0.40* | **0.47** | *0.40* | **0.48** |  | *65-62 | - | - | - | - | - |
| 151 | **0.43** | *0.39* | **0.46** | *0.40* | **0.47** |  | 61 | 0.26 | *0.37* | *0.41* | 0.32 | 0.27 |
| 150 | *0.36* | 0.30 | *0.35* | 0.32 | *0.34* |  | 60 | **0.48** | **0.54** | **0.58** | **0.47** | **0.46** |
| *149-147 | - | - | - | - | - |  | 59 | **0.52** | **0.61** | **0.62** | **0.55** | **0.53** |
| 146 | -0.24 | *-0.40* | *-0.39* | *-0.35* | *-0.33* |  | 58 | **0.55** | **0.63** | **0.66** | **0.58** | **0.57** |
| 145 | -0.24 | *-0.42* | **-0.43** | *-0.40* | *-0.37* |  | 57 | **0.53** | **0.62** | **0.67** | **0.58** | **0.59** |
| 144 | -0.22 | *-0.35* | *-0.38* | *-0.38* | *-0.33* |  | 56 | **0.51** | **0.61** | **0.65** | **0.55** | **0.55** |
| *143-139 | - | - | - | - | - |  | 55 | **0.50** | **0.55** | **0.63** | **0.50** | **0.51** |
| 138 | *0.39* | *0.36* | **0.45** | *0.38* | *0.41* |  | 54 | **0.47** | **0.51** | **0.53** | *0.42* | **0.45** |
| 137 | *0.36* | *0.39* | **0.46** | *0.42* | **0.43** |  | 53 | **0.50** | **0.53** | **0.51** | **0.43** | **0.48** |
| 136 | **0.43** | *0.41* | **0.54** | **0.43** | **0.46** |  | 52 | **0.47** | **0.54** | **0.49** | **0.46** | **0.51** |
| 135 | *0.35* | *0.37* | **0.44** | 0.32 | *0.40* |  | 51 | **0.51** | **0.53** | **0.50** | **0.46** | **0.51** |
| 134 | *0.36* | *0.37* | *0.42* | 0.32 | **0.43** |  | 50 | **0.56** | **0.52** | **0.47** | **0.47** | **0.50** |
| 133 | 0.26 | 0.29 | 0.32 | 0.26 | *0.35* |  | 49 | **0.46** | *0.35* | 0.32 | *0.37* | *0.39* |
| *132-129 | - | - | - | - | - |  | 48 | **0.46** | 0.28 | 0.28 | 0.32 | 0.32 |
| 128 | 0.19 | 0.29 | 0.25 | 0.29 | *0.39* |  | 47 | **0.47** | *0.35* | *0.34* | *0.37* | *0.35* |
| 127 | *0.34* | *0.38* | 0.30 | 0.33 | **0.44** |  | 46 | **0.46** | *0.34* | 0.32 | *0.35* | *0.37* |
| 126 | *0.40* | *0.41* | *0.40* | **0.43** | **0.49** |  | 45 | **0.49** | 0.31 | 0.31 | *0.39* | *0.38* |
| 125 | *0.42* | **0.47** | **0.52** | **0.47** | **0.51** |  | 44 | *0.41* | 0.32 | 0.26 | *0.36* | *0.35* |
| 124 | **0.45** | **0.46** | **0.50** | **0.45** | **0.53** |  | 43 | *0.40* | 0.28 | 0.18 | 0.28 | 0.31 |
| 123 | 0.27 | *0.37* | 0.32 | *0.34* | **0.45** |  | *42-30 | - | - | - | - | - |
| 122 | 0.22 | *0.37* | 0.31 | *0.36* | **0.43** |  | 29 | 0.27 | 0.28 | 0.22 | *0.34* | *0.36* |
| *121-108 | - | - | - | - | - |  | 28 | 0.27 | 0.29 | 0.22 | *0.35* | *0.37* |
| 107 | *-0.40* | -0.30 | -0.24 | -0.27 | *-0.33* |  | 27 | 0.22 | 0.27 | 0.20 | *0.33* | *0.36* |
| 106 | **-0.44** | *-0.39* | -0.31 | -0.32 | *-0.39* |  | 26 | 0.22 | 0.28 | 0.22 | *0.34* | *0.36* |
| 105 | **-0.43** | *-0.35* | -0.28 | -0.28 | *-0.36* |  | 25 | 0.29 | 0.32 | 0.28 | *0.38* | *0.40* |
| 104 | *-0.35* | -0.27 | -0.22 | -0.22 | -0.29 |  | 24 | *0.35* | *0.38* | *0.34* | **0.43** | **0.45** |
| 103 | -0.30 | -0.27 | -0.24 | -0.26 | -0.30 |  | 23 | *0.40* | **0.42** | *0.38* | **0.45** | **0.48** |
| 102 | -0.31 | -0.32 | -0.32 | *-0.39* | *-0.41* |  | 22 | *0.35* | *0.42* | *0.34* | *0.40* | **0.47** |
| 101 | -0.30 | *-0.36* | *-0.38* | **-0.49** | **-0.48** |  | 21 | *0.36* | **0.45** | *0.36* | *0.40* | **0.46** |
| 100 | -0.24 | -0.32 | *-0.40* | **-0.45** | *-0.40* |  | 20 | *0.41* | **0.43** | *0.37* | *0.41* | **0.46** |
| 99 | -0.32 | **-0.44** | **-0.47** | **-0.49** | **-0.45** |  | 19 | **0.46** | **0.45** | *0.40* | **0.44** | **0.48** |
| 98 | -0.26 | **-0.43** | **-0.46** | **-0.50** | **-0.43** |  | 18 | **0.48** | **0.46** | *0.41* | **0.44** | **0.48** |
| 97 | -0.19 | *-0.38* | *-0.41* | **-0.44** | *-0.38* |  | 17 | **0.44** | **0.44** | *0.39* | *0.42* | **0.49** |
| 96 | -0.20 | *-0.35* | *-0.40* | **-0.49** | *-0.39* |  | 16 | **0.45** | **0.47** | *0.42* | **0.48** | **0.51** |
| 95 | 0.00 | -0.25 | -0.25 | *-0.36* | -0.28 |  | 15 | **0.48** | **0.44** | **0.45** | **0.49** | **0.52** |
| 94 | -0.04 | -0.29 | -0.25 | *-0.35* | -0.26 |  | 14 | **0.53** | **0.45** | **0.49** | **0.49** | **0.52** |
| *93-85 | - | - | - | - | - |  | 13 | **0.48** | **0.45** | **0.49** | **0.50** | **0.53** |
| 84 | -0.29 | -0.30 | -0.21 | -0.29 | *-0.33* |  | 12 | **0.45** | **0.44** | **0.48** | **0.48** | **0.53** |
| 83 | -0.27 | -0.30 | -0.22 | -0.31 | -0.33 |  | 11 | **0.49** | **0.45** | **0.49** | **0.46** | **0.53** |
| 82 | -0.28 | -0.30 | -0.23 | -0.33 | *-0.35* |  | 10 | **0.47** | **0.46** | **0.46** | **0.45** | **0.51** |
| 81 | -0.26 | -0.31 | -0.27 | *-0.38* | *-0.38* |  | 9 | *0.36* | *0.34* | *0.38* | *0.38* | *0.41* |
| 80 | -0.31 | *-0.36* | *-0.33* | **-0.43** | **-0.45** |  | 8 | *0.41* | *0.33* | *0.39* | *0.41* | **0.42** |
| 79 | *-0.36* | *-0.40* | *-0.37* | **-0.44** | **-0.47** |  | 7 | *0.42* | *0.33* | *0.37* | *0.36* | *0.40* |
| 78 | **-0.44** | **-0.44** | *-0.39* | **-0.43** | **-0.48** |  | 6 | *0.39* | 0.31 | *0.38* | *0.39* | *0.42* |
| 77 | **-0.48** | **-0.45** | *-0.40* | *-0.42* | **-0.48** |  | 5 | *0.36* | *0.38* | **0.47** | **0.49** | **0.50** |
| 76 | **-0.50** | **-0.47** | *-0.41* | **-0.44** | **-0.50** |  | 4 | **0.46** | *0.41* | **0.45** | **0.46** | **0.55** |
| 75 | **-0.52** | **-0.48** | **-0.43** | **-0.46** | **-0.53** |  | 3 | *0.37* | 0.30 | *0.36* | *0.38* | **0.44** |
| 74 | **-0.54** | **-0.51** | **-0.47** | **-0.51** | **-0.58** |  | 2 | *0.33* | 0.23 | *0.35* | 0.31 | *0.36* |
| 73 | **-0.56** | **-0.57** | **-0.54** | **-0.58** | **-0.65** |  | 1 | *0.38* | 0.26 | *0.35* | *0.36* | *0.38* |

*: Ranges of consecutive spectral signals for which non-significant correlations were observed for all target species are omitted.

## **Supplementary Table S4.** Results as in Tables S2-S4, but for 4 perennial plants: *Acanthus mollis* (Aca), *Ampelodesmos mauritanicus* (Amp), *Festuca drymeia* (Fes), and *Hedera helix* (Hed).

| **^13^C NMR**  **(ppm)** | **Correlation with root growth** | | | |  | **^13^C NMR**  **(ppm)** | **Correlation with root growth** | | | |
| --- | --- | --- | --- | --- | --- | --- | --- | --- | --- | --- |
|  | **Aca** | **Amp** | **Fes** | **Hed** |  |  | **Aca** | **Amp** | **Fes** | **Hed** |
| *190-182 | - | - | - | - |  | 89 | 0.11 | *0.37* | *0.35* | 0.10 |
| 181 | -0.18 | *-0.37* | *-0.39* | -0.27 |  | 88 | 0.11 | *0.39* | *0.40* | 0.12 |
| *180-158 | - | - | - | - |  | 87 | 0.12 | *0.36* | *0.40* | 0.11 |
| 157 | 0.06 | 0.19 | 0.00 | *0.39* |  | 86 | 0.11 | 0.31 | *0.39* | 0.09 |
| 156 | 0.11 | 0.28 | 0.15 | **0.50** |  | 85 | 0.10 | 0.30 | *0.36* | 0.08 |
| 155 | 0.15 | *0.39* | 0.22 | **0.52** |  | 84 | 0.12 | *0.35* | *0.39* | 0.10 |
| 154 | 0.26 | **0.55** | 0.30 | **0.56** |  | 83 | 0.14 | *0.35* | *0.38* | 0.13 |
| 153 | 0.29 | **0.51** | 0.31 | **0.56** |  | 82 | 0.19 | 0.32 | *0.37* | 0.18 |
| 152 | *0.42* | **0.55** | **0.44** | **0.53** |  | 81 | 0.18 | 0.29 | *0.34* | 0.21 |
| 151 | 0.32 | *0.37* | 0.32 | *0.37* |  | *80-66 | - | - | - | - |
| 150 | 0.22 | 0.30 | 0.27 | *0.38* |  | 65 | 0.21 | *0.36* | **0.46** | 0.16 |
| 149 | 0.22 | **0.43** | 0.30 | **0.46** |  | 64 | 0.32 | *0.40* | **0.51** | 0.21 |
| 148 | *0.34* | *0.36* | 0.30 | **0.54** |  | 63 | *0.34* | *0.40* | **0.48** | 0.32 |
| 147 | 0.16 | 0.14 | -0.16 | **0.51** |  | 62 | **0.44** | *0.38* | *0.38* | *0.41* |
| 146 | -0.06 | -0.08 | *-0.35* | **0.46** |  | 61 | **0.47** | 0.31 | 0.23 | *0.34* |
| 145 | -0.10 | -0.13 | *-0.34* | *0.40* |  | 60 | *0.40* | 0.25 | 0.22 | 0.21 |
| 144 | -0.02 | -0.01 | -0.26 | *0.38* |  | 59 | *0.33* | 0.23 | 0.16 | 0.16 |
| *143-134 | - | - | - | - |  | 58 | *0.34* | 0.26 | 0.18 | 0.18 |
| 133 | 0.21 | 0.17 | -0.05 | *0.36* |  | 57 | *0.41* | 0.30 | 0.21 | 0.21 |
| *132-125 | - | - | - | - |  | 56 | *0.36* | 0.23 | 0.16 | 0.20 |
| 124 | *0.37* | 0.18 | 0.25 | 0.26 |  | *55-49 | - | - | - | - |
| 123 | 0.32 | 0.19 | 0.27 | 0.30 |  | 48 | -0.09 | *-0.35* | -0.15 | 0.00 |
| 122 | *0.34* | 0.17 | 0.14 | *0.35* |  | *47-45 | - | - | - | - |
| 121 | *0.39* | 0.16 | 0.13 | **0.43** |  | 44 | -0.17 | *-0.39* | -0.29 | -0.14 |
| 120 | *0.37* | 0.10 | 0.01 | **0.43** |  | 43 | -0.18 | *-0.40* | *-0.36* | -0.13 |
| 119 | 0.29 | 0.21 | 0.13 | *0.33* |  | 42 | -0.18 | **-0.43** | *-0.41* | -0.13 |
| 118 | *0.39* | *0.42* | 0.19 | **0.45** |  | 41 | -0.29 | **-0.55** | **-0.46** | -0.23 |
| 117 | *0.39* | *0.41* | 0.20 | **0.48** |  | 40 | -0.27 | **-0.48** | **-0.44** | -0.22 |
| 116 | 0.32 | **0.43** | 0.26 | **0.44** |  | 39 | -0.28 | **-0.46** | **-0.47** | -0.26 |
| 115 | 0.30 | **0.46** | *0.39* | *0.42* |  | 38 | *-0.39* | **-0.51** | **-0.56** | -0.27 |
| 114 | 0.30 | **0.46** | **0.48** | *0.42* |  | 37 | **-0.43** | **-0.50** | **-0.53** | -0.33 |
| 113 | 0.30 | **0.47** | **0.44** | *0.42* |  | 36 | **-0.45** | **-0.48** | **-0.47** | *-0.40* |
| 112 | *0.38* | **0.51** | **0.43** | **0.42** |  | 35 | *-0.39* | **-0.44** | *-0.38* | *-0.40* |
| 111 | **0.43** | **0.53** | **0.44** | *0.41* |  | 34 | -0.31 | *-0.34* | -0.23 | *-0.37* |
| 110 | 0.32 | **0.55** | **0.44** | *0.37* |  | 33 | -0.31 | -0.30 | -0.22 | *-0.36* |
| 109 | 0.23 | **0.45** | *0.41* | *0.36* |  | 32 | -0.31 | -0.32 | -0.30 | *-0.40* |
| 108 | 0.14 | 0.33 | *0.36* | 0.21 |  | 31 | -0.29 | *-0.34* | -0.31 | *-0.40* |
| *107-106 | - | - | - | - |  | 30 | -0.25 | -0.30 | -0.30 | *-0.36* |
| 105 | 0.14 | *0.34* | 0.30 | 0.18 |  | 29 | -0.23 | -0.28 | -0.28 | *-0.35* |
| 104 | 0.26 | *0.39* | *0.36* | 0.25 |  | 28 | -0.26 | -0.31 | -0.31 | *-0.36* |
| *103-102 | - | - | - | - |  | 27 | -0.30 | -0.33 | *-0.34* | *-0.38* |
| 101 | 0.22 | 0.14 | 0.18 | *0.35* |  | 26 | -0.30 | -0.32 | *-0.33* | *-0.41* |
| 100 | 0.18 | 0.27 | 0.20 | *0.36* |  | 25 | -0.27 | -0.29 | -0.28 | *-0.42* |
| 99 | 0.13 | 0.26 | 0.11 | *0.40* |  | 24 | -0.17 | -0.24 | -0.21 | *-0.35* |
| 98 | 0.03 | 0.19 | 0.06 | *0.34* |  | *23-1 | - | - | - | - |
| *97-90 | - | - | - | - |  |  |  |  |  |  |

*: Ranges of consecutive spectral signals for which non-significant correlations were observed for all target species are omitted.

## **Supplementary Table S5.** Results as in Tables S2-S5, but for 5 woody plants: *Alnus glutinosa* (Aln), *Pinus halepensis* (Pin), *Populus nigra* (Pop), *Quercus ilex* (Que), and *Robinia pseudoacacia* (Rob).

| **^13^C NMR**  **(ppm)** | **Correlation with root growth** | | | | |  | **^13^C NMR**  **(ppm)** | **Correlation with root growth** | | | | |
| --- | --- | --- | --- | --- | --- | --- | --- | --- | --- | --- | --- | --- |
|  | **Aln** | **Pin** | **Pop** | **Que** | **Rob** |  |  | **Aln** | **Pin** | **Pop** | **Que** | **Rob** |
| *190-178 | - | - | - | - | - |  | 121 | 0.23 | 0.11 | 0.18 | 0.12 | *0.34* |
| 177 | -0.13 | -0.26 | -0.08 | *-0.35* | -0.29 |  | *120-117 | - | - | - | - | - |
| *176-157 | - | - | - | - | - |  | 116 | 0.15 | 0.06 | 0.07 | *0.34* | 0.25 |
| 156 | 0.23 | 0.13 | 0.15 | *0.39* | 0.31 |  | 115 | 0.14 | 0.11 | 0.08 | *0.36* | *0.37* |
| 155 | 0.24 | 0.14 | 0.20 | **0.44** | *0.39* |  | 114 | 0.19 | 0.10 | 0.16 | *0.39* | **0.43** |
| 154 | 0.26 | 0.11 | 0.30 | *0.39* | **0.47** |  | 113 | 0.09 | 0.09 | 0.16 | *0.35* | *0.40* |
| 153 | 0.23 | 0.12 | *0.34* | *0.35* | **0.55** |  | 112 | 0.11 | 0.11 | 0.18 | *0.35* | *0.39* |
| 152 | 0.29 | 0.11 | *0.38* | 0.31 | **0.53** |  | 111 | 0.11 | 0.08 | 0.21 | *0.34* | *0.40* |
| 151 | 0.16 | 0.03 | 0.29 | 0.24 | **0.45** |  | *110-100 | - | - | - | - | - |
| 150 | 0.15 | 0.08 | 0.23 | 0.30 | *0.42* |  | 99 | 0.00 | 0.01 | -0.04 | *0.38* | 0.04 |
| 149 | 0.19 | 0.10 | 0.21 | *0.38* | *0.38* |  | 98 | -0.09 | -0.03 | -0.02 | *0.38* | 0.08 |
| 148 | 0.14 | 0.09 | 0.25 | *0.38* | *0.36* |  | *97-61 | - | - | - | - | - |
| 147 | 0.04 | -0.03 | 0.01 | *0.33* | 0.05 |  | 60 | 0.13 | -0.02 | *0.37* | -0.10 | 0.23 |
| 146 | -0.06 | 0.06 | -0.12 | *0.35* | -0.05 |  | 59 | 0.13 | 0.01 | *0.38* | -0.11 | 0.27 |
| *145-136 | - | - | - | - | - |  | 58 | 0.16 | 0.03 | *0.41* | -0.10 | 0.32 |
| 135 | 0.23 | 0.03 | 0.26 | 0.02 | *0.40* |  | 57 | 0.16 | 0.04 | *0.41* | -0.13 | 0.31 |
| 134 | 0.23 | 0.10 | *0.36* | 0.07 | **0.43** |  | 56 | 0.15 | 0.00 | *0.41* | -0.12 | 0.27 |
| *133-125 | - | - | - | - | - |  | 55 | 0.12 | -0.05 | *0.39* | -0.12 | 0.22 |
| 124 | 0.23 | 0.11 | *0.41* | 0.08 | *0.42* |  | *54-5 | - | - | - | - | - |
| 123 | *0.34* | 0.05 | 0.27 | 0.07 | *0.42* |  | 4 | 0.02 | 0.10 | *0.33* | -0.12 | 0.31 |
| 122 | 0.29 | 0.07 | 0.19 | 0.06 | 0.33 |  | *3-1 | - | - | - | - | - |

*: Ranges of consecutive spectral signals for which non-significant correlations were observed for all target species are omitted

## **Supplementary Table S6.** List of target plants, fungi and bacteria used in the bioassays. For plants, we reported the weight of 1,000 seeds (source: database of the Kew Royal Botanic Gardens, UK) and the duration of the root proliferation experiment.

| **Kingdom** | **Functional group** | **Species** | **Weight of 1,000 seeds (g)** | **Experiment duration (days)** |
| --- | --- | --- | --- | --- |
| Bacteria | Saprotroph | *Bacillus subtilis* |  |  |
|  | Plant pathogen | *Erwinia carotovora* |  |  |
|  | Coliform, pathogen | *Escherichia coli* |  |  |
|  | Saprotroph, | *Lysobacter gummosus* |  |  |
|  | Saprotroph, pathogen | *Pseusomonas fluorescens* |  |  |
|  | Plant pathogen | *Rhizobium radiobacter* |  |  |
| Fungi | Saprotroph | *Aspergillus niger* |  |  |
|  | Plant pathogen | *Botrytis cinerea* |  |  |
|  | Saprotroph, mycoparasitic | *Trichoderma* *harzianum* |  |  |
|  | Plant pathogen, saprotroph | *Ganoderma lucidum* |  |  |
|  | Saprotroph | *Mucor* sp. |  |  |
|  | Saprotroph | *Umbelopsis ramanniana* |  |  |
| Plants | Annual | *Arabidopsis thaliana* | 0.02 | 21 |
|  | Annual | *Lepidium sativum* | 2.17 | 14 |
|  | Annual | *Lycopersicon esculentum* | 1.97 | 14 |
|  | Annual | *Trifolim pratense* | 1.42 | 14 |
|  | Annual | *Triticum durum* | 41.24 | 14 |
|  | Perennial | *Acanthus mollis,* | 223.18 | 28 |
|  | Perennial | *Ampelodesmos mauritanicus* | 4.58 | 28 |
|  | Perennial | *Festuca drymeia* | 2.63 | 28 |
|  | Perennial | *Hedera helix* | 31.72 | 28 |
|  | Woody tree | *Alnus glutinosa* | 2.02 | 28 |
|  | Woody tree | *Pinus halepensis* | 22.05 | 28 |
|  | Woody tree | *Populus nigra* | 0.81 | 28 |
|  | Woody tree | *Quercus ilex* | 2321.85 | 28 |
|  | Woody tree | *Robinia pseudoacacia* | 19.21 | 28 |

## **Supplementary Fig. S1.** Results of bioassays with six bacteria (a), six fungi (b) and fourteen plant species (c) growing on either undecomposed (0 d) or decomposing (180 d) litter. Growth is expressed as percent compared to the controls (nutrient broth, PDA and water for bacteria, fungi, and plants, respectively). Target species are ranked by decreasing growth over undecomposed litter. Data for each bar refer to mean ± standard deviation of 18 litter types. Asterisks indicate age-dependent significant differences for each target species (***, *p* < 0.001; **, *p* < 0.01; *, *p* < 0.05; n.s., *p* > 0.05), according to Tuckey's HSD post-hoc tests for the interactive effects of litter age and target species from GLMMs models in Table 2.

##
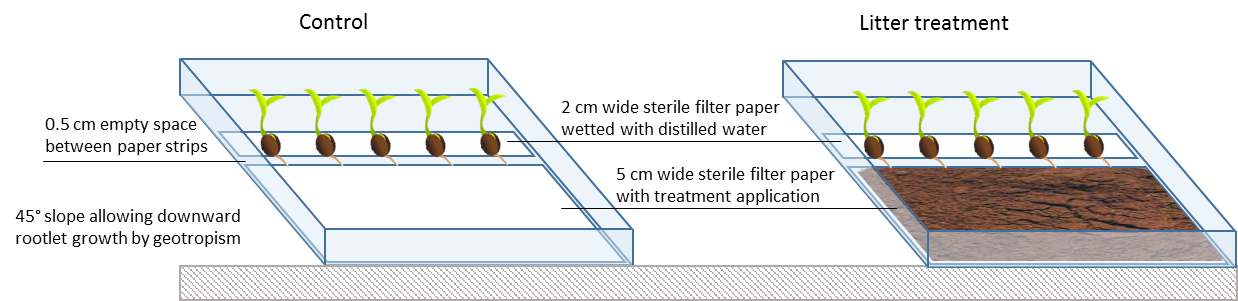


## **Supplementary Fig. S2.** Scheme of Petri dishes (12 x 12 x 1.5 cm) with experimental setup for plant bioassay, showing control (i.e. sterile distilled water) and litter treatments. The empty space between the two paper strips used for either seed placement or treatment application prevented treatment interference on seed germination, while ensuring rootlet proliferation in the litter layer. A total of 5180 Petri dishes were prepared (14 plant target species × 36 litter types plus untreated control × 10 replicates), arranged following a totally randomized design in a growing room under controlled temperature (16±2°C night and 22±2°C day) and relative humidity (55%) conditions, and covered with opaque sheets when roots were not under observation.
